# *Arabidopsis* CaM1 and CaM4 Promote Nitric Oxide Production and Salt Resistance by Inhibiting *S*-Nitrosoglutathione Reductase via Direct Binding

**DOI:** 10.1371/journal.pgen.1006255

**Published:** 2016-09-29

**Authors:** Shuo Zhou, Lixiu Jia, Hongye Chu, Dan Wu, Xuan Peng, Xu Liu, Jiaojiao Zhang, Junfeng Zhao, Kunming Chen, Liqun Zhao

**Affiliations:** 1 Hebei Key Laboratory of Molecular and Cellular Biology, Key Laboratory of Molecular and Cellular Biology of the Ministry of Education, College of Life Sciences, Hebei Normal University, Hebei Collaboration Innovation Center for Cell Signaling, Shijiazhuang, China; 2 State Key Laboratory of Crop Stress Biology in Arid Areas, College of Life Sciences, Northwest A & F University, Yangling, China; Duke University, UNITED STATES

## Abstract

Salt is a major threat to plant growth and crop productivity. Calmodulin (CaM), the most important multifunctional Ca^2+^ sensor protein in plants, mediates reactions against environmental stresses through target proteins; however, direct proof of the participation of CaM in salt tolerance and its corresponding signaling pathway *in vivo* is lacking. In this study, we found that *AtCaM1* and *AtCaM4* produced salt-responsive *CaM* isoforms according to real-time reverse transcription-polymerase chain reaction analyses; this result was verified based on a phenotypic analysis of salt-treated loss-of-function mutant and transgenic plants. We also found that the level of nitric oxide (NO), an important salt-responsive signaling molecule, varied in response to salt treatment depending on *AtCaM1* and *AtCaM4* expression. GSNOR is considered as an important and widely utilized regulatory component of NO homeostasis in plant resistance protein signaling networks. *In vivo* and *in vitro* protein-protein interaction assays revealed direct binding between AtCaM4 and *S*-nitrosoglutathione reductase (GSNOR), leading to reduced GSNOR activity and an increased NO level. Overexpression of *GSNOR* intensified the salt sensitivity of *cam4* mutant plants accompanied by a reduced internal NO level, whereas a *gsnor* deficiency increased the salt tolerance of *cam4* plants accompanied by an increased internal NO level. Physiological experiments showed that CaM4-GSNOR, acting through NO, reestablished the ion balance to increase plant resistance to salt stress. Together, these data suggest that AtCaM1 and AtCaM4 serve as signals in plant salt resistance by promoting NO accumulation through the binding and inhibition of GSNOR. This could be a conserved defensive signaling pathway in plants and animals.

## Author Summary

Calmodulins (CaMs) are a highly conserved protein family in eukaryotes. They are known to be important for plant tolerance against external stimuli. Here we described a new molecular function of the *Arabidopsis thaliana* CaMs in response to salinity. We reported that two *CaM* isoforms *AtCaM1* and *AtCaM4*, which encode the same protein, were stimulated in a salt stress-dependent manner. Also, we showed that AtCaM4 and AtCaM1 directly bound to *S*-nitrosoglutathione reductase (GSNOR) and then inhibited its activity so as to enhance internal level of nitric oxide (NO). Finally, we found that AtCaM4-GSNOR through NO regulated ion absorption to confer salt resistance. Thus, our study presents a novel model for salt stress-signaling pathway. In mammalian cells, CaM participates in a wide variety of processes, including neurotransmission, vasodilation, and immune defense, also by regulating the production of NO through NO synthase. Therefore, our findings support the idea of a common evolutionary origin of this defense system in higher eukaryotes.

## Introduction

Soil salinization is one of the most important ecological crises today. High salinity alters normal plant growth and development via osmotic stress and ion toxicity. Elucidation of the mechanisms by which plants recognize and respond to salt stress is of great interest to plant biologists seeking to understand cellular signaling mechanisms and to apply that knowledge to generate plants that can be grown in saline soil.

Although plants, as sessile organisms, cannot escape from salt stress, they have developed sophisticated adaptive mechanisms that enable them to perceive and respond to a saline environment. As a countermeasure, calcium (Ca^2+^), a universal second messenger in eukaryotes, acts on downstream Ca^2+^ sensor proteins by influencing their localization, conformation, and function, and it induces a series of physiological and biochemical reactions to resist these adverse environmental conditions [1]. In plants, the three largest families of Ca^2+^ sensor proteins are calmodulins (CaMs) and CaM-like proteins [2], Ca^2+^-dependent protein kinases (CDPKs) [3,4], and calcineurin B-like proteins (CBLs) [5,6].

Among these proteins, some members of the CDPK and CBL families in *Arabidopsis thaliana* (hereafter, Arabidopsis) have been shown to participate in salt signal transduction. For example, *AtCPK3* expression, which is triggered by salt, is required for MAPK-independent salt-stress acclimation in Arabidopsis [7]. AtCPK6 is a functionally redundant, positive regulator of salt/drought stress tolerance [8]. Previous studies of CBL function indicate that CBL4 (Salt Overly Sensitive 3, AtSOS3) improves the salt tolerance of plants by interacting with SOS2, and that it regulates the expression of *AtSOS1*, which encodes a plasma membrane Na^+^/H^+^ antiporter [9–11]. CaM is also thought to be involved in salt stress signaling. The expression of CaM in sweet potato (*Ipomoea batatas* L.) is induced by NaCl [12]. A specific CaM isoform mediates salt-induced Ca^2+^ signaling through the activation of a MYB transcriptional activator, resulting in salt tolerance in plants [13]. Overexpression of *GmCaM4* in soybean (*Glycine max* L.) enhances plant resistance to pathogens and tolerance to salt stress [14]. However, direct proof of the participation of CaM in salt tolerance and its corresponding signaling pathway *in vivo* is lacking. Additional studies are needed to obtain new insight into the salt signaling network.

CaM is the most important multifunctional Ca^2+^ sensor in eukaryotes. The structure and function of plant CaMs are similar to those of animal and yeast CaMs; however, plant genomes contain multiple CaM genes that encode identical CaM isoforms (about 6–12) [15,16]. The existence of similar amino acid sequences among isoforms is a distinguishing characteristic of higher plants [17]. The activation of specific CaM isoforms by special stimulating factors initiates a series of responsive reactions; thus, the diversity among CaM isoforms is an important factor leading to specific CaM signaling pathways. Given this, identifying which CaM isoforms are responsive to salt was a primary focus of the present study. CaM is composed of soluble single-chain proteins, each consisting of two globular domains connected by an α-helical linker. Each of the two globular head domains consists of two helix-loop-helix motifs (EF hands), each of which binds a single Ca^2+^ ion. Ca^2+^ binding to CaM induces the exposure of hydrophobic clefts that can then interact with downstream targets [18]. CaMs are non-enzymatic proteins; however, Ca^2+^ binding promotes the attachment of CaM to the short peptide sequence of a special target protein, modulating its activity; this may influence cell division, growth, development, and stress reactions [19–21]. The CaM targets in plants include metabolic enzymes, kinases, phosphatases, transcription factors (TFs), channels, pumps, cytoskeletal proteins, and proteins of unknown function; the list is still growing thanks to the use of interactive proteomic analysis [22,23]. Thus, a second focus of this study was to explore the downstream targets activated by salt-induced CaM isoforms in the salt signaling pathway. By addressing these two issues, we hope to promote in-depth and systematic studies of the molecular mechanisms by which CaM induces salt adaptation in plants.

The model plant Arabidopsis has been widely used in studies of plant growth, resistance to adverse stimuli, and hormonal/environmental factor-induced signal conducting systems, including Ca^2+^-mediated signal transduction systems. Given the wealth of knowledge that exists about Arabidopsis, it is the preferred material for studies of CaM-mediated signal transduction. The determination of the sequence of the Arabidopsis genome enabled the identification of candidate genes encoding CaM proteins; however, only seven of these genes actually encode CaMs (named *AtCaM1–7*). Intriguingly, these seven distinct genes encode only four CaM isoforms: *AtCaM1*/*4*, *AtCaM2*/*3*/*5*, *AtCaM6*, and *AtCaM7* [2]. A number of studies have shown that different CaM isoforms respond to unique stimuli. For example, *AtCaM3* and *AtCaM7* regulate the expression of genes related to cold and light responses, respectively [24,25]. We recently showed that AtCaM3 functions in the induction of thermotolerance, which is dependent on increased heat shock TF DNA-binding activity and heat shock protein accumulation [26–28]. Therefore, it is possible that certain or several CaM isoforms are salt-responsive factor(s) that induce salt responses in plants by interacting with a downstream target protein.

Nitric oxide (NO), which functions as an important messenger in multiple biological processes in plants, is induced by numerous biotic and abiotic stresses to mediate resistance responses [29,30]. It also induces salt resistance in two ecotypes of reed (*Phragmites communis* Trin.) by increasing the potassium (K^+^)/sodium (Na^+^) ratio [31]. In Arabidopsis, NOA1-dependent NO production in plant cells is associated with salt tolerance [32]. NIA/ NR/NOA1-dependent NO production supports heme oxygenase 1 expression in the modulation of plant salt tolerance [33]. These data suggest that NO plays a crucial role in salt-stress signaling; however, the precise mechanism remains elusive.

In plants, NO is produced mainly through two different enzymatic pathways. In the first pathway, NO is generated by nitrate reductase through the successive reduction of nitrate to nitrite and then to NO. In the second pathway, L-Arg (with oxygen and NADPH) is converted to NO and citrulline by the action of NO synthase (NOS); however, the actual existence and identity of NOS in plants is currently unresolved [34]. On the other hand, cells possess various mechanisms for removing NO. For example, NO reacts with glutathione (GSH) to form *S*-nitrosylated glutathione (GSNO), which is then metabolized by the enzyme GSNO reductase (GSNOR). In Arabidopsis, GSNOR is a cytosolic protein that is encoded by a single copy gene (At5g43940) [35].

As signaling molecules, CaM and NO play important roles in eliciting plant resistance reactions. Studies of CaM and NO in plants and animals have shown significant overlap in their individual pathways; however, it remains controversial which is upstream of the other. In mammalian cells, CaM was reported to bind and activate NOS isozymes with physiological relevance [36]. The two pairs of EF hands in CaM play different roles in the binding and activation of mammalian inducible NOS, constitutive NOSs, endothelial NOS, and neuronal NOS [37,38]. A FRET study clarified some of the observed similarities and differences in the Ca^2+^-dependent/independent interactions between CaM and NOS isozymes [39]. Interestingly, the opposite situation exists in plants; CaM is considered to be a downstream factor of NO. Indeed, we reported that NO acts upstream of AtCaM3 in thermotolerance in Arabidopsis seedlings [27]. Also, the AtNOA1-dependent production of NO plays a crucial role in extracellular CaM-induced stomatal closure [40]. As yet, the relationship between CaM and NO is obscure in plants exposed to salt injury. In this study, we used the model plant Arabidopsis to explore the CaM signaling system under conditions of salt stress. Our results show that AtCaM1 and AtCaM4 are involved in salt resistance through the binding and subsequent inhibition of GSNOR, which enhances NO accumulation.

## Results

### The Relationship Between *AtCaM1* and *AtCaM4* and Salt Resistance

**Fig 1:**
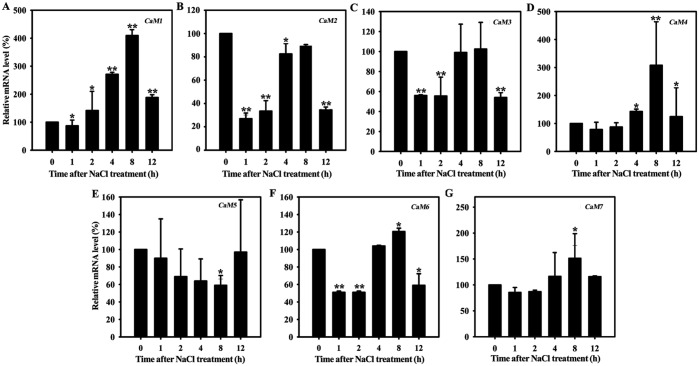
Salt exposure influences *AtCaM* expression. RT-qPCR analysis of *AtCaM1* (A), *AtCaM2* (B), *AtCaM3* (C), *AtCaM4* (D), *AtCaM5* (E), *AtCaM6* (F), and *AtCaM7* (G) transcription in 7-day-old wild-type seedlings grown in 0.5× MS liquid medium with 50 mM NaCl for 0–12 h. The *18S rRNA* was used as an internal control. The experiments were repeated three times with similar results. Each data point represents the mean ± standard deviation (SD, n = 3). Asterisks indicate a significant difference relative to 0 h (Student’s t-test, *t*-test, *P < 0.05 and **P < 0.01).

CaM, as the major Ca^2+^ sensor in plants, is involved in the responses of plants to a wide range of environmental stresses, including salt stress [41,42]. To determine which CaM isoform responds to salt, we first examined the expression of *AtCaM1* (At5g37780), *AtCaM2* (At2g27030), *AtCaM3* (At3g56800), *AtCaM4* (At1g66410), *AtCaM5* (At2g41110), *AtCaM6* (At5g21274), and *AtCaM7* (At3g43810) using reverse transcriptase quantitative polymerase chain reaction (RT-qPCR). Total RNA samples were prepared from wild-type seedlings treated with 50 mM NaCl. The *AtCaM1* and *AtCaM4* expression levels increased initially, reaching their highest values at 8 h (410 and 308% of the control level, respectively); they then decreased, but remained higher than in the control at 12 h (Fig 1A and 1D). The expression of the other genes showed no obvious regular variation (Fig 1B, 1C and 1E–1G). Thus, we reached the preliminary conclusion that out of all of the *AtCaM* genes investigated, *AtCaM1* and *AtCaM4*, which encode the same protein [2], likely function in the response of Arabidopsis to salt.

To confirm the role of *AtCaM1* and *AtCaM4* in salt stress tolerance, we compared the phenotypes of wild-type and mutant seedlings treated with or without salt stress. Due to the lack of an available T-DNA insertion mutant of *AtCaM1* from the Arabidopsis Biological Resource Center (ABRC), we selected specific base sites (S1A Fig) to construct an artificial microRNA vector and then introduced it into wild-type and T-DNA insertion mutant *cam4* (GABI_309E09) [26] plants to generate RNA interference (RNAi) transgenic lines. Next, four lines, *cam1-1*, *cam1-2*, *cam1/4-1*, and *cam1/4-2*, were selected for salt sensitivity analysis. No clear morphological difference was observed between 4-week-old wild-type and mutant plants under normal growth conditions (S1 Fig).

**Fig 2:**
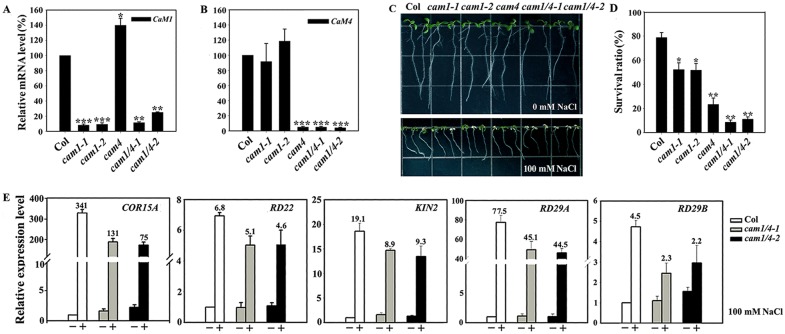
*AtCaM1* and *AtCaM4* contribute to salt tolerance. (A, B) RT-qPCR analysis of *AtCaM1* (A) and *AtCaM4* (B) transcription in wild-type and mutant plants. The experiments were repeated three times with similar results. Each data point represents the mean ± SD (n = 3). Asterisks indicate a significant difference relative to Columbia (Col) (Student’s t-test, *t*-test, *P < 0.05, **P < 0.01, and ***P < 0.001). (C) Salt stress sensitivity of 7-day-old seedlings grown in 0.5× MS medium with or without 100 mM NaCl. The experiments were repeated three times with similar results. (D) Survival ratios of the seedlings after salt treatment. Those seedlings with still green cotyledons were scored as survivors. Each data point represents the mean ± standard error (SE, n = 30). Asterisks indicate a significant difference relative to Col (Student’s *t*-test, *P < 0.05 and **P < 0.01). (E) RT-qPCR analysis of salt-responsive gene expression in wild-type, *cam1/4-1*, and *cam1/4-2* seedlings. The experiments were repeated three times with similar results. Each data point represents the mean ± SD (n = 3). The numbers at the top indicate the fold change in mRNA level after the salt challenge.

RT-qPCR analysis revealed dramatically reduced expression of *AtCaM1* in *cam1-1*, *cam1-2*, *cam1/4-1*, and *cam1/4-2* plants (7, 9, 13, and 23% of the control level, respectively), and nearly complete disruption of *AtCaM4* in *cam4*, *cam1/4-1*, and *cam1/4-2* plants (Fig 2A and 2B). However, deficiency in *AtCaM4* slightly stimulated the expression of *AtCaM1* (Fig 2A). Further, no obvious variation was found in the transcript levels of *AtCaM2*, *AtCaM3*, *AtCaM5*, *AtCaM6*, and *AtCaM7* in these plants, indicating that a deficiency in *AtCaM1* or *AtCaM4* expression did not influence the expression of the other *AtCaM* genes (S2 Fig). Phenotypic observation indicated that the mutant seedlings were indistinguishable from wild-type seedlings under normal growth conditions. However, the effects of salt on the survival of the wild-type and mutant seedlings differed (Fig 2C). Following growth in medium containing 100 mM NaCl for 7 days [43], the survival ratios of the *cam1-1*, *cam1-2*, and *cam4* mutants (55, 56, and 23%, respectively) were lower than that of wild type (79%). Double mutant (*cam1/4-1* and *cam1/4-2*) seedlings showed greater sensitivity to salt stress than did the single mutant seedlings. Also, the survival ratio of the *cam1/4-1* seedlings (12%) was lower than that of the *cam1/4-2* seedlings (14%), consistent with their observed transcript levels (Fig 2D).

Next, we identified unique bases in *AtCaM1* and *AtCaM4* through a comparison to other *CaM* genes (S3A Fig) in order to produce RNAi transgenic lines. Two lines, *cam1/4-3* and *cam1/4-4*, were selected for analysis. No obvious morphological difference was observed among 4-week-old wild-type and mutant plants under normal growth conditions (S3B Fig). RT-qPCR analysis showed that the transcript levels of *AtCaM1* and *AtCaM4* were greatly decreased in the *cam1/4-3* and *cam1/4-4* plants, especially in *cam1/4-*3 (S3C and S3D Fig). Under salt stress, the survival ratios of the *cam1/4-3* and *cam1/4-4* seedlings were significantly reduced (31 and 25%, respectively) compared with that of wild-type seedlings; the greater reduction in the *cam1/4-*3 plants mirrors the observed decrease in mRNA expression (S4 Fig). We next detected salt-induced genes in Arabidopsis and found that their expression was dramatically induced by NaCl in wild-type, but to a lesser extent in *cam1/4-1* and *cam1/4-2*, seedlings (Fig 2E), further indicating that *AtCaM1* and *AtCaM4* function in salt tolerance.

Seven distinct genes in Arabidopsis encode AtCaMs [2]; we utilized T-DNA insertion mutants of *cam2* (SALK_114166) and *cam3* (SALK_001357), with disrupted expression of *AtCaM2* and *AtCaM3*, respectively [26], to examine the roles of other *CaM* genes in salt tolerance. Under normal growth conditions, no obvious phenotypic difference was detected among the wild-type, *cam2*, and *cam3* seedlings. The same result was obtained following salt treatment: the survival ratios of *cam2* and *cam3* mutant seedlings were similar to that of wild-type seedlings (S5 Fig). These observations suggest that other *AtCaM* genes do not function in salt resistance.

### Effect of *AtCaM4* on the Salt Sensitivity of *cam1/4-1* Mutant Plants

To further test whether the salt sensitivity of the mutant plants resulted from the loss of *AtCaM1* and *AtCaM4*, *AtCaM4* complementation lines (*cam1/4-1* + *AtCaM4*, 4COM), and *AtCaM1* and *AtCaM4* overexpression lines (ecotype Columbia [WT] + *AtCaM1*, 1OE; ecotype Columbia [WT] + *AtCaM4*, 4OE) were generated and confirmed by RT-qPCR and reverse transcription polymerase chain reaction (RT-PCR), respectively (S6, S8 and S9 Figs). In the *AtCaM4* complementation lines (4COM1 and 4COM2), the *AtCaM1* mRNA level was rescued to a near wild-type level, suggesting ineffective RNAi (S6A and S6B Fig). Under normal growth conditions, none of the transgenic lines showed a mutant phenotype compared with wild type (S6, S8 and S9 Figs). When subjected to salt stress for 7 days, the *AtCaM4* complementation lines exhibited enhanced survival, similar to wild type (S7 Fig), providing genetic proof of the involvement of AtCaM1 and AtCaM4 in salt resistance.

However, no significant morphological difference was detected between the wild-type and *AtCaM1*- and *AtCaM4*-overexpressing lines (1OE1 and 1OE2, and 4OE1 and 4OE2, respectively) under conditions of salt stress (S8C and S9C Figs).

### Effects of Salt Stress on NO Accumulation and Survival in Wild-Type and Mutant Seedlings

In mammalian cells, CaM is thought to bind and thus activate NOS isozymes to stimulate NO production. NO, as a signaling molecule, plays an important role in the salt stress signaling pathway in Arabidopsis seedlings [32]. Thus, it is reasonable to assume that *AtCaM1* and *AtCaM4* mediate salt resistance by regulating NO metabolism.

**Fig 3:**
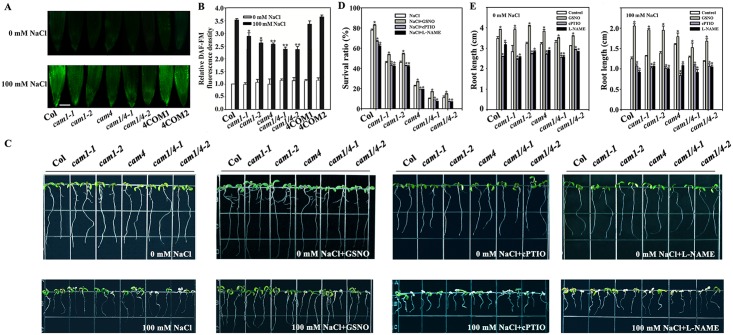
*AtCaM1* and *AtCaM4* are positive regulators of salt-mediated NO accumulation. (A) NO accumulation in the roots of 7-day-old wild-type, *cam1-1*, *cam1-2*, *cam4*, *cam1/4-1*, *cam1/4-2*, 4COM1 and 4COM2 seedlings grown in 0.5× MS liquid medium with or without 100 mM NaCl for 24 h was detected by DAF-FM DA staining. The experiments were repeated three times with similar results. Bar = 50 μm. (B) Relative DCF fluorescence densities in the roots. Each data point represents the mean ± SE (n = 20). Asterisks indicate a significant difference relative to Col (Student’s *t*-test, *P < 0.05 and **P < 0.01). (C) Salt stress sensitivity of 5-day-old seedlings grown in 0.5× MS medium with or without 100 mM NaCl supplemented with 50 μM GSNO, 100 μM c-PTIO or 150 μM L-NAME for another 2 days. The experiments were repeated three times with similar results. (D) Survival ratios of the seedlings after salt treatment. Each data point represents the mean ± SE (n = 30). Asterisks indicate a significant difference relative to NaCl (Student’s t-test, *P < 0.05). (E) Root lengths of the seedlings with or without 100 mM NaCl. Each data point represents the mean ± SE (n = 30). *P < 0.05 (Student’s *t*-test).

Accordingly, we examined intracellular NO formation in wild-type, *cam1-1*, *cam1-2*, *cam4*, *cam1/4-1*, and *cam1/4-2* plants and in two *AtCaM4* complementation lines at the seedling stage. 4-Amino-5-methylamino-2',7'-difluorofluorescein diacetate (DAF-FM DA) was selected for use as a fluorescent probe for NO because it is highly specific for NO and does not react with other reactive oxygen species. DAF-FM DA permeated the membrane and was transformed by intracellular esterases into 4-amino-5-methylamino-2',7'-difluorofluorescein (DAF-FM), which reacts with NO to create a highly fluorescent triazole compound [44]. A special NO scavenger 2-phenyl-4,4,5,5-tetramethyl-imidazoline-1-oxyl-3-oxide (cPTIO) decreased the fluorescence density depending on its concentration, indicating DAF-FM DA was the special probe for NO (S10 Fig). Fluorescence analysis revealed that the NO levels were relatively stable in the seedlings under normal growth conditions. However, the NO level was remarkably increased in the presence of NaCl and varied depending on the expression of *AtCaM1* and *AtCaM4*; it was increased by 260% in wild-type seedlings, which is greater than in the *cam1-1*, *cam1-2*, *cam4*, *cam1/4-1*, and *cam1/4-2* mutant seedlings (the lowest values were obtained from the *cam1/4-1* and *cam1/4-2* double mutants; 72 and 81%, respectively). However, the NO level was nearly completely rescued in the *AtCaM4* complementation lines (Fig 3A and 3B). By combining these data with the results of our salt tolerance analysis (Fig 2), we might conclude that the salt sensitivity of *cam1-1*, *cam1-2*, *cam4*, *cam1/4-1*, and *cam1/4-2* was due to the low endogenous NO level.

To further confirm the effects of NO on the salt sensitivity of the mutant plants, we examined the effects of NO donor and inhibitors on their survival. Exogenous application of 50 μM GSNO, as NO donor, increased the root lengths of the plants under both normal and high-salt conditions, and it increased the survival ratios of the mutant seedlings under high-salt conditions. Whereas exogenous application of 100 μM cPTIO or 150 μM *N*^*G*^-nitro-L-arginine-methyl ester (L-NAME, a NO synthase inhibitor) showed the adverse effects on them (Fig 3C to 3E), indicating that NO acts as a downstream mediator of AtCaM1 and AtCaM4 in salt tolerance.

### Examination of Direct Binding Between AtCaM4 and GSNOR

CaM, as a central signaling molecule, likely confers salt tolerance by binding directly to a specific target protein. Therefore, we next sought to identify interacting proteins of AtCaM4 (and AtCaM1, omitted) to gain insight into the roles of CaM in salt signaling.

**Fig 4:**
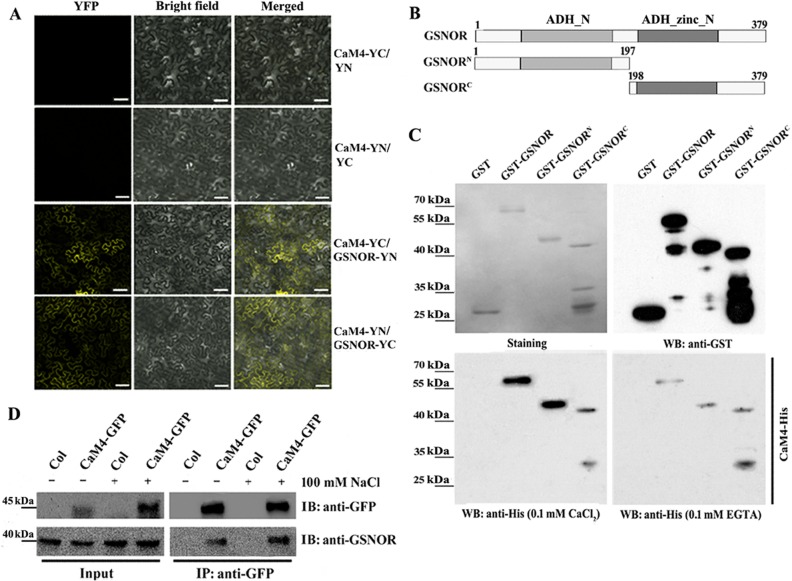
AtCaM4 binds to GSNOR in response to salt stress. (A) *In planta* BiFC assay of the CaM4 interaction with GSNOR in tobacco leaves. The C-terminal half of YFP was fused to CAM4 (CaM4-YC) and GSNOR (GSNOR-YC), and the N-terminal half of YFP was fused to GSNOR (GSNOR-YN) and CAM4 (CaM4-YN). The expression of CaM4-YC/YN and CaM4-YN/YC was used as a control. Bar = 50 μm. (B) Model of the GSNOR N- and C-terminal deletions for the overlay assay. (C) *In vitro* overlay assay of the CaM4 interaction with GSNOR. GST and GST-GSNOR fusion proteins were expressed in *E*. *coli*. Recombinant proteins were analyzed by Western blotting (WB) using anti-GST antibodies. The overlay assay was performed using CaM4-His in the presence of 0.1 mM CaCl_2_ or 0.1 mM EGTA. (D) Co-IP assays showing the interaction between CaM4 and GSNOR. CaM4-GFP was expressed from the native *CaM4* promoter. Extracted proteins were incubated with anti-GFP agarose beads. Total and immunoprecipitated proteins were analyzed by immunoblotting using anti-GSNOR and -GFP antibodies.

A number of experimental approaches have been employed in previous studies to identify CaM-interacting proteins [13, 45]. Herein, we carried out experiments to explore what AtCaM4-binding protein(s) regulates NO homeostasis in salt-treated plants. According to a structural analysis conducted using WebLab ViewerLite (Accelrys, San Diego, CA), GSNOR (among NO metabolism-related proteins; see the Introduction) harbors important binding elements for the paired EF hands in AtCaM4 (S11 Fig). Thus, we performed three assays to test whether AtCaM4 is a substrate of GSNOR. A bimolecular fluorescence complementation (BiFC) assay [46] in tobacco (*Nicotiana tabacum*) leaves showed that negative combinations, including CaM4-YN/YC (in which the N-terminal followed by the C-terminal half of yellow fluorescent protein [YFP] was fused to CAM4) and CaM4-YC/YN (in which the C-terminal followed by the N-terminal half of YFP was fused to CAM4), did not produce any detectable fluorescence, while the co-expression of CaM4-YN (in which the N-terminal half of YFP was fused to CAM4) and GSNOR-YC (in which the C-terminal half of YFP was fused to GSNOR) or CaM4-YC (in which the C-terminal half of YFP was fused to CAM4) and GSNOR-YN (in which the N-terminal half of YFP was fused to GSNOR) produced strong YFP signals, which were mainly localized to the plasma membrane of the cotransformed tobacco epidermal cells (Fig 4A). Furthermore, we performed a domain deletion analysis (see SMART, http://smart.embl-heidelberg.de) by overlay assay (Fig 4B). To map the CaM-binding domain of GSNOR, we created a series of GSH *S*-transferase (GST) fusion constructs containing the full-length cDNA and two serial deletion mutants. Our results show that GST-tagged full-length GSNOR, the N-terminal fragment of GSNOR, and the C-terminal fragment of GSNOR all interacted with CaM-His when Ca^2+^ was present in the medium, whereas GST alone did not interact with AtCaM4. However, the binding became weaker in the presence of 0.1 mM EGTA (a Ca^2+^ chelator) (Fig 4C), indicating that AtCaM4 binds to GSNOR at multiple important regions (S11 Fig) in a Ca^2+^-dependent manner (Fig 4B). The interaction of CaM4 and GSNOR *in vivo* was confirmed using co-immunoprecipitation (co-IP) assays. Proteins were extracted from Arabidopsis harboring GSNOR and *CaM4*_*pro*_:*CaM4-green fluorescent protein* (*GFP*) constructs and used for co-IP assays. As shown in Fig 4D, the immunoprecipitation of CaM4 with anti-GFP agarose conjugate yielded a co-IP band corresponding to GSNOR that was labeled with anti-GSNOR antibodies; moreover, the signal intensity was increased by exposure to NaCl. Taken together, these *in vivo* and *in vitro* protein-protein interaction assays indicate a direct interaction between CaM4 and GSNOR.

**Fig 5:**
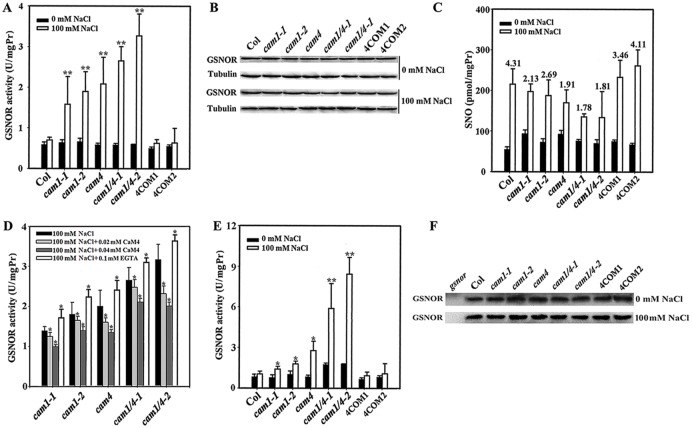
AtCaM4 decreases GSNOR activity in response to salt stress. (A, E) GSNOR activity of total protein (A) or purified protein (E) from 7-day-old wild-type, *cam1-1*, *cam1-2*, *cam4*, *cam1/4-1*, *cam1/4-2*, 4COM1 and 4COM2 seedlings grown in 0.5 × MS medium with or without 100 mM NaCl. The experiments were repeated three times with similar results. Each data point represents the mean ± SD (n = 3). Asterisks indicate a significant difference relative to 0 mM NaCl (Student’s *t*-test, *P < 0.05 and **P < 0.01). (B) Immunoblot analysis of GSNOR expression in the seedlings. Tubulin was used as an internal control. The experiments were repeated three times; the results indicate similar trends in protein accumulation. (C) SNO content in the seedlings grown in 0.5× MS medium with or without 100 mM NaCl. The experiments were repeated three times with similar results. Each data point represents the mean ± SD (n = 3). The numbers at the top indicate the fold change in SNO level after the salt challenge. (D) GSNOR activity in 7-day-old *cam1-1*, *cam1-2*, *cam4*, *cam1/4-1*, and *cam1/4-2* seedling after addition of 0.1 mM EGTA, 0.02 or 0.04 mM CaM4-GST in the reaction mixture. The experiments were repeated three times with similar results. Each data point represents the mean ± SD (n = 3). Asterisks indicate a significant difference relative to 100 mM NaCl (Student’s *t*-test, *P < 0.05). (F) Immunoblot analysis of purified GSNOR from total protein with anti-GSNOR. *gsnor* seedlings was used as a negative control. The experiments were repeated three times; the results indicate similar trends in protein accumulation.

What is the effect of this binding on GSNOR activity? We measured the GSNOR activity in wild-type, *cam1-1*, *cam1-2*, *cam4*, *cam1/4-1*, and *cam1/4-2* plants as well as in two *AtCaM4* complementation lines with total protein and purified GSNOR protein from the seedlings. Our data indicate no clear difference among the seedlings in terms of GSNOR activity under normal conditions. However, the level of GSNOR activity from total protein was greatly increased by NaCl in the *cam1-1*, *cam1-2*, *cam4*, *cam1/4-1*, and *cam1/4-2* mutant seedlings (it was highest for *cam1/4-1* and *cam1/4-2*), whereas it was only slightly increased in the wild-type, 4COM1, and 4COM2 seedlings (Fig 5A). Immunoblotting showed that GSNOR expression did not vary noticeably in the seedlings treated with and without salt (Fig 5B), indicating that AtCaM4 inhibited GSNOR activity directly but did not greatly influence its expression. Levels of NO-related metabolite *S*-nitrosothiols (SNOs) *in vivo* are controlled by NO synthesis and by GSNO turnover, which is mainly performed by GSNOR [47]. Thus, we measured the variation of SNO content in the seedlings after NaCl treatment. Our data indicate that total SNO levels were only slightly increased in the *cam1-1*, *cam1-2*, *cam4*, *cam1/4-1*, and *cam1/4-2* mutant seedlings (it was lowest for *cam1/4-1* and *cam1/4-2*), whereas it was greatly increased in the wild-type, 4COM1, and 4COM2 seedlings (Fig 5C), in an opposite changing manner as GSNOR activity (Fig 5A), implying that increased GSNOR activity in the mutant seedlings (especially for *cam1/4-1* and *cam1/4-2*) inhibited SNO accumulation. Exogenous application of CaM4-GST fusion inhibited GSNOR activity in a concentration-dependent manner, whereas exogenous application of EGTA enhanced it in the *cam1-1*, *cam1-2*, *cam4*, *cam1/4-1*, and *cam1/4-2* mutant seedlings (Fig 5D), providing the straight evidence for AtCaM4 inhibition of GSNOR activity. The activity of the same content of purified GSNOR protein from these seedlings (Fig 5F) also showed the same changing manner (Fig 5E) as that of total protein (Fig 5A), further indicating that AtCaM4 inhibition of GSNOR activity was not due to the variation of GSNOR expression. Together, these data show that AtCaM4 bound to GSNOR directly and influenced its activity under salt stress; thus, GSNOR is a target of AtCaM4 in the salt signaling pathway.

Unexpectedly, an *in vitro* experiment using CaM4-His and GST-GSNOR fusions, which were expressed in *Escherichia coli* (*E*. *coli*) and purified (S12A Fig), showed that CaM4 had no obvious effect on GSNOR activity (S12B Fig). A plausible explanation for this strange phenomenon could be the need of a proper conformation or additional posttranslational modification of both proteins.

### GSNOR Negatively Regulates the NO Level and Salt Tolerance in Plants

An analysis by RT-qPCR indicated that *GSNOR* expression was slightly stimulated by salt treatment (S13A Fig). β-Glucuronidase (GUS) staining of *GSNOR*_*pro*_:*GUS* transgenic plants showed that the *GSNOR* promoter drove expression ubiquitously (S13B Fig), suggesting that it has extensive activities in plants. To determine the subcellular localization of GSNOR and AtCaM4, *35S*_*pro*_:*GSNOR-GFP* and *35Spro*:*CaM4-GFP* fusions were introduced into tobacco leaves, respectively; similarly, both of GFP fluorescence was found to be localized mainly in the cell membrane, cytoplasm, and nucleus (S13C and S14 Figs).

**Fig 6:**
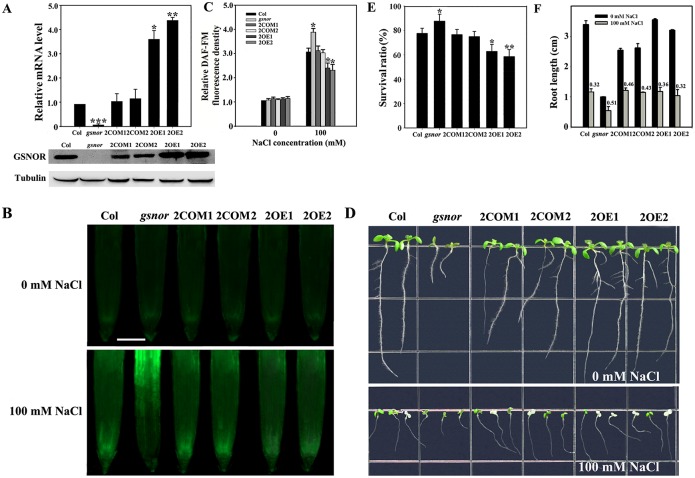
*GSNOR* acts as a negative regulator of salt tolerance. (A) RT-qPCR and immunoblot analyses of *GSNOR* expression in wild-type, *gsnor*, 2COM1, 2COM2, 2OE1 and 2OE2 plants. The experiments were repeated three times with similar results. Each data point represents the mean ± SD (n = 3). Asterisks indicate a significant difference relative to Col (Student’s *t*-test, *P < 0.05, **P < 0.01, and ***P < 0.001). (B) NO accumulation in the roots of 7-day-old seedlings. The experiments were repeated three times with similar results. Bar = 50 μm. (C) Relative DCF fluorescence densities in the roots. Each data point represents the mean ± SE (n = 20). Asterisks indicate a significant difference relative to Col (Student’s *t*-test, *P < 0.05 and **P < 0.01). (D) Salt stress sensitivity of the seedlings in 0.5× MS medium with or without 100 mM NaCl. The experiments were repeated three times with similar results. (E) Survival ratios of the seedlings after salt treatment. Each data point represents the mean ± SE (n = 30). Asterisks indicate a significant difference relative to Col (Student’s *t*-test, *P < 0.05 and **P < 0.01). (F) Root lengths of the seedlings with or without 100 mM NaCl. Each data point represents the mean ± SE (n = 30). The numbers at the top indicate the fold change in root length after the salt challenge.

GSNOR is believed to be an important and widely utilized regulatory component of NO homeostasis in plant resistance protein signaling networks [45, 48–52]. The T-DNA mutant *gsnor* (CS66012, also named *hot5-2* [53]), which carries an insertion in exon 1, was obtained from the ABRC. To analyze the physiological role of GSNOR in plants under salt stress, we assayed the NO level and salt tolerance in wild-type and *gsnor* plants, and in two complementation lines (*gsnor* + *GSNOR*, 2COM) and two overexpression lines (ecotype Columbia [WT] + *GSNOR*, 2OE), which were verified by RT-qPCR and immunoblot analyses for transcript and protein accumulation (Fig 6A). Thereafter, fluorescence analysis revealed no obvious change in NO among the seedlings under normal conditions. However, the NO level increased clearly under 100 mM NaCl treatment and was far higher in the *gsnor* seedlings than in the wild-type seedlings, fully restored in the two *gsnor* complementation lines (2COM1 and 2COM2), and reversed in the two *gsnor*-overexpression lines (2OE1 and 2OE2) (Fig 6B and 6C). Additionally, the *gsnor* seedlings were small under both normal and high-salt conditions; however, their survival ratio was 14% higher than that of wild-type seedlings when grown on NaCl-containing medium. This situation was restored in the two complementation lines and reversed in the two overexpression lines (Fig 6D and 6E), implying that GSNOR contributes to salt sensitivity via inhibition of the endogenous NO level in plants. Simultaneously, the root length of the *gsnor* seedlings was less reduced compared to that of wild-type seedlings in the existence of NaCl. This situation was partially restored in the complementation and overexpression lines depending on their internal NO levels (Fig 6F), implying NO stimulation of root growth.

### The Relationship between AtCaM4 and GSNOR in Response to Salt Stress

**Fig 7:**
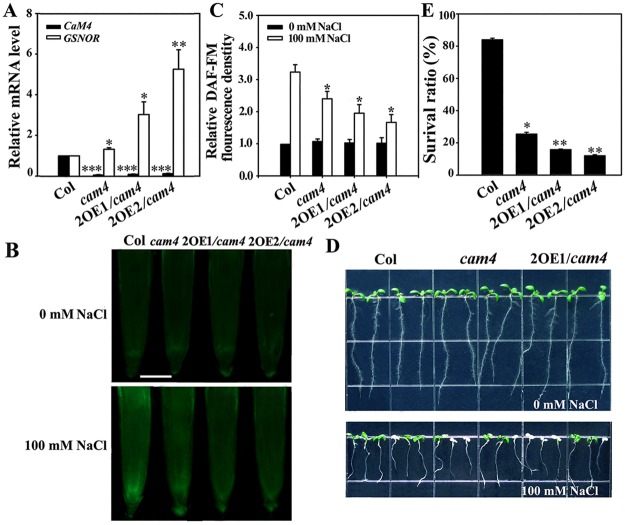
*GSNOR* overexpression inhibits NO accumulation and enhances salt sensitivity in *cam4*. (A) RT-qPCR analysis of *AtCaM4* and *GSNOR* transcription in wild-type, *cam4*, 2OE1/*gsnor* and 2OE2/*gsnor* plants. The experiments were repeated three times with similar results. Each data point represents the mean ± SD (n = 3). Asterisks indicate a significant difference relative to Col (Student’s *t*-test, *P < 0.05, **P < 0.01, and ***P < 0.001). (B) NO accumulation in the roots of 7-day-old seedlings. The experiments were repeated three times with similar results. Bar = 50 μm. (C) Relative DCF fluorescence densities in the roots. Each data point represents the mean ± SE (n = 20). Asterisks indicate a significant difference relative to Col (Student’s *t*-test, *P < 0.05 and **P < 0.01). (D) Salt stress sensitivity of the seedlings in 0.5× MS medium with or without 100 mM NaCl. The experiments were repeated three times with similar results. (E) Survival ratios of the seedlings after salt treatment. Each data point represents the mean ± SE (n = 30). Asterisks indicate a significant difference relative to Col (Student’s *t*-test, *P < 0.05 and **P < 0.01).

To examine the underlying mechanism of AtCaM1/4- and GSNOR-induced salt tolerance in Arabidopsis, we obtained *GSNOR*-overexpressing transgenic lines in a *cam4* background (2OE/*cam4*) and compared their NO levels and survival. RT-qPCR revealed stronger exogenous *GSNOR* expression in the 2OE1/*cam4* and 2OE2/*cam4* lines than in wild type (Fig 7A). These two lines did not show obvious variation in the level of NO compared with *cam4* under normal growth conditions. However, under 100 mM NaCl treatment, the NO levels and survival ratios of the two transgenic lines were lower than those of *cam4* (survival ratios of 17 and 15% versus 23%, respectively), indicating that overexpression of *GSNOR* further reduced the NO level and strengthened the salt sensitivity of the *cam4* mutant (Fig 7B–7E).

**Fig 8:**
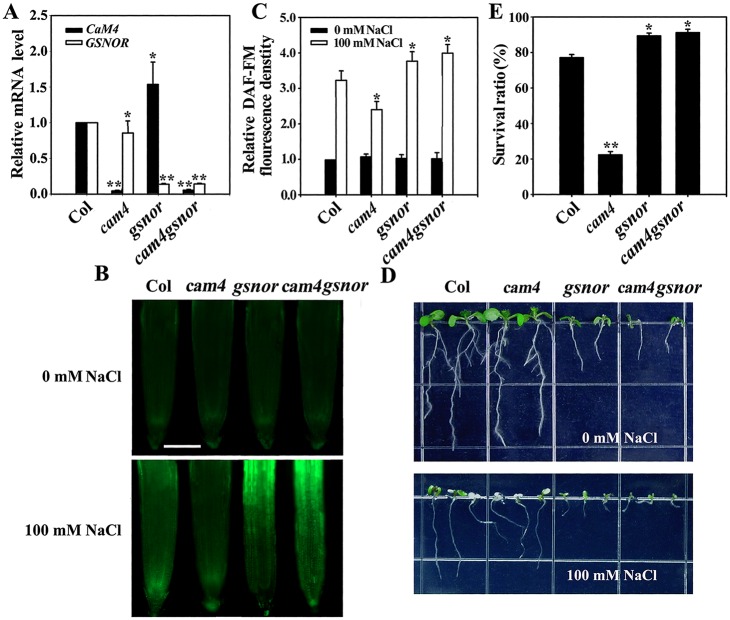
*GSNOR* disruption enhances NO accumulation and inhibits salt sensitivity in *cam4*. (A) RT-qPCR analysis of *AtCaM4* and *GSNOR* transcription in wild-type, *cam4*, *gsnor*, and *cam4gsnor* plants. The experiments were repeated three times with similar results. Each data point represents the mean ± SD (n = 3). Asterisks indicate a significant difference relative to Col (Student’s *t*-test, *P < 0.05, and **P < 0.01). (B) NO accumulation in the roots of 7-day-old seedlings. The experiments were repeated three times with similar results. Bar = 50 μm. (C) Relative DCF fluorescence densities in the roots. Each data point represents the mean ± SE (n = 20). Asterisks indicate a significant difference relative to Col (Student’s *t*-test, *P < 0.05). (D) Salt stress sensitivity of the seedlings in 0.5× MS medium with or without 100 mM NaCl. The experiments were repeated three times with similar results. (E) Survival ratios of the seedlings after salt treatment. Each data point represents the mean ± SE (n = 30). Asterisks indicate a significant difference relative to Col (Student’s *t*-test, *P < 0.05 and **P < 0.01).

We also compared the NO levels and survival of wild-type, *cam4*, *gsnor*, and *cam4gsnor* double mutant (deficient in *CaM4* and *GSNOR* transcription) seedlings (Fig 8A). Under normal growth conditions, the NO levels in the seedlings were not obviously different; however, they were increased by NaCl. Surprisingly, the NO level in *cam4gsnor* was increased compared with that in *cam4* and similar to that in *gsnor* (Fig 8B and 8C). Regardless of whether NaCl was present, the *cam4gsnor* double mutant seedlings showed reduced shoot and root growth, like the *gsnor* seedlings. After salt treatment, the survival ratio changed in a manner similar to the NO level: the survival ratio of *cam4gsnor* was higher than that of wild type and similar to that of *gsnor* (Fig 8D and 8E).

Taking these results together, we might conclude that AtCaM1 and AtCaM4 confer salt tolerance by mediating NO accumulation through GSNOR.

### AtCaM4 and GSNOR Modulate Ion Absorption in Arabidopsis Seedlings

When plants are exposed to NaCl, cellular ion homeostasis may be impaired. Under saline conditions, tolerant plants typically maintain high K^+^ and low Na^+^ levels in the cytosol via the compartmentalization of Na^+^ into vacuoles and/or extrusion to the external medium and the accumulation of K^+^ in the cytoplasm [54,55].

**Fig 9:**
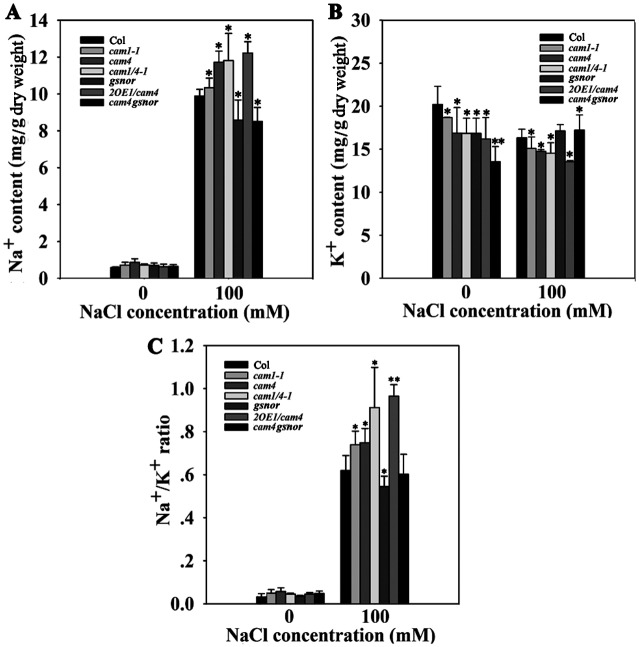
AtCaM4 influences ion accumulation in response to salt stress through GSNOR. The (A) Na^+^ content, (B) K^+^ content, and (C) Na^+^/K^+^ ratio in 7-day-old wild-type, *cam1-1*, *cam4*, *cam1/4-1*, *2OE1/cam4*, and *cam4gsnor* seedlings grown in 0.5× MS liquid medium with or without 100 mM NaCl for 24 h. The experiments were repeated three times with similar results. Each data point represents the mean ± SD (n = 3). Asterisks indicate a significant difference relative to Col in each group (Student’s *t*-test, *P < 0.05 and **P < 0.01).

It was previously reported that NO functions as a second messenger in reestablishing ion homeostasis to resist salt stress in reed calluses (*P*. *communis* Trin.) [31] and Arabidopsis seedlings [32]. In the present study, we tested the effects of CaM4-GSNOR on the NO-mediated regulation of ion absorption in Arabidopsis seedlings exposed to excessive salt. The inhibition of K^+^ absorption and stimulation of Na^+^ absorption, as well as a decreased K^+^/Na^+^ ratio, were observed in *cam1-1*, *cam4*, and *cam1/4-1* mutant plants compared with wild type (especially in *cam1/4-1*), though the opposite situation was detected in the *gsnor* mutant. This trend was intensified in the *GSNOR*-overexpressing transgenic line 2OE1/*cam4* but was partially rescued in the *cam4gsnor* double mutant compared to *cam4* (Fig 9), suggesting that AtCaM4 enhances K^+^ absorption and inhibits Na^+^ absorption through GSNOR in plants under salt stress.

## Discussion

### *AtCaM1* and *AtCaM4* Confer Salt Resistance in Arabidopsis Seedlings

CaMs are predicted to function in response to a rise in the cytoplasmic concentration of Ca^2+^ in many physiological processes in plants and animals [20]. Considering the many processes in which they are involved, their diverse subcellular localization patterns, and their assorted responses to various environmental stimuli, each CaM isoform may have a specific function [56]; for example, *AtCaM3* is involved in the induction of cold- and heat-responsive genes [25, 26], while *AtCaM7* is involved in the induction of light-responsive genes [24]. Currently, the role of CaM in the salt signaling pathway is elusive, greatly limiting our knowledge of plant adaptation to salt stress.

The present study demonstrates the involvement of AtCaM1 and AtCaM4 in salt stress signaling. In salt-treated plants, AtCaM1 and AtCaM4 act as second messengers; they bind GSNOR and reduce its activity so as to elevate the endogenous NO level and reestablish cellular ion homeostasis. Thus, AtCaM1 and AtCaM4 promote salt resistance in Arabidopsis seedlings.

CaM is regarded as a signaling molecule that contributes to salt tolerance; however, its signaling pathway is unknown. To address this, two key elements must be identified: salt-responsive CaM isoforms and their corresponding target protein(s). In Arabidopsis, only seven genes encode real CaM proteins [2]. Therefore, we first examined their expression under salt stress. RT-qPCR analysis showed that *AtCaM1* and *AtCaM4* transcript accumulation varied regularly under saline conditions (Fig 1). The disruption of *AtCaM1* and *AtCaM4* resulted in poor salt tolerance in *cam1-1*, *cam1-2*, *cam4*, and double mutant plants depending on their transcript levels (Fig 2). The knockdown of both *AtCaM1* and *AtCaM4* exacerbated the salt sensitivity of the plants compared to the knockdown of only *AtCaM1* (S4 Fig), suggesting the positive role of these genes in salt resistance. Rescued *AtCaM4* expression in *AtCaM1/4-1* nearly totally restored the salt sensitivity of the plants (S7 Fig), providing genetic support for this hypothesis.

A loss of *AtCaM1* and *AtCaM4* impaired salt-responsive signaling, as evidenced by the significant decrease in the fold changes of salt-induced genes in RNAi plants compared with wild-type plants (Fig 2E). Taken together, these observations suggest that *AtCaM1* and *AtCaM4* each contribute to salt resistance and that their functions do not overlap.

Genetic studies have been invaluable in improving our understanding of the role of CaMs in angiosperms. However, the high level of sequence identity among members of the CaM family and the likelihood of functional redundancy have complicated this approach. Thus, we examined the expression and function of other CaM isoforms. Our results indicate that a loss of *AtCaM1* and *AtCaM4* transcription did not influence the expression of other CaM isoforms in plants (S2 Fig). Further, the deletion of *AtCaM2* and *AtCaM3* did not obviously modulate plant salt sensitivity (S5 Fig). These data suggest that *AtCaM1* and *AtCaM4* are the salt-responsive CaM isoforms; other CaM isoforms may not affect salt tolerance in the seedlings.

We also observed a strange phenomenon: the overexpression of *AtCaM1* or *AtCaM4* did not enhance salt tolerance (S8 and S9 Figs), possibly due to the natural high abundance of AtCaMs and the amount of interacting protein (i.e. GSNOR; Fig 5B).

### AtCaM4 Regulates NO Production in Response to Salinity

In mammalian cells, CaM, a ubiquitous 17-kDa cytosolic protein, is a major cellular Ca^2+^ sensor that rapidly regulates intracellular processes through its coordinated activity with more than 50 intracellular proteins, including NOS [57]. Previous studies have shown that CaM participates in a wide variety of processes, including neurotransmission, vasodilation, and immune defense, by regulating the production of NO through NOS [58]. In plants, a NO synthesis-related enzyme is stimulated by salt to enhance the internal NO level and initiate plant defensive reactions [31,32]. Thus, we hypothesized that CaMs mediate the NO level to initiate plant responses to salt stress.

As shown in Fig 3A and 3B, the internal NO level increased depending on the expression levels of *AtCaM1* and *AtCaM4* in response to salt stress. Also, the NO donor GSNO increased the salt tolerance of *AtCaM1* and *AtCaM4* mutant plants to a level near that of wild type but the NO inhibitors cPTIO and L-NAME decreased it (Fig 3C and 3D), providing further proof for this hypothesis. NO, which functions as an important messenger in multiple biological processes in plants, is induced by numerous biotic and abiotic stresses to mediate resistance responses, however, it relationship with CaM remained to be elucidated. Therefore, our study implies a common defense system in plant. Among the three major families of Ca^2+^ sensors in terrestrial plants, only CaMs co-exist in plants and animals. Thus, these findings support the idea of a common pathway of this defense system in higher eukaryotes.

### GSNOR Is the Target Protein of AtCaM4 in Response to Salinity

Identifying the specific target (or substrate) of a CaM isoform is a key step in understanding the functions of CaM in plant signaling. The specific target of a CaM protein must exhibit two functional characteristics: it must bind to CaM, and its activity must subsequently change. A transient expression study with protoplasts indicated that the Ca^2+^/CaM complex functions as a negative regulator of the activity of the rice (*Oryza sativa*) CAMTA/SR protein Os-CBT [59]. The expression of two CaM-binding TFs in bean is induced in response to incompatible pathogens and elicitors of plant defense responses, suggesting a role for CaM-binding TFs in plant defense [60]. A loss-of-function mutant of a CaM-binding phosphatase (PP7) exhibited reduced heat tolerance, whereas its overexpression, which increased heat shock protein expression, conferred thermotolerance [61]. Since this phosphatase interacts with a heat shock TF (HSF1), it is likely that Ca^2+^/CaM modulates the activity of HSF1 through PP7.

Accordingly, we first sought to identify AtCaM4-binding proteins involved in NO metabolism in plants under salt stress. In mammalian cells, CaMs bind NOS isozymes directly. A bioinformatics analysis did not predict the interaction of AtCaM4 and NO synthesis-related proteins, including NO-associated protein 1, nitrate reductase 1, and nitrate reductase 2 [34]; however, it suggested the binding of AtCaM4 to GSNOR (S11 Fig), which degrades GSNO, a stable and mobile NO pool, to reduce the overall accumulation of NO in plant cells. *In vivo* and *in vitro* assays revealed direct binding between AtCaM4 and GSNOR (Fig 4A–4D), which could be inhibited by a Ca^2+^ chelator, indicating that this binding was initiated by enhancement of the cytoplasmic Ca^2+^ concentration. N- and C-terminal fragments of GSNOR interacted with CaM4, indicating that two or more elements in GSNOR bind the paired EF hands in AtCaM4 (Fig 4C), consistent with the predicted model (S11 Fig). Further, the binding of AtCaM4 to GSNOR was reinforced in the presence of NaCl (Fig 4D), indicating a possible role in the response of plants to salt stress.

Next, we showed that CaM4 inhibited GSNOR activity according to its expression level but had no great effect on GSNOR expression (Fig 5). We also found that deficiency in *CaM4* led to slightly lower *GSNOR* mRNA level (Fig 8A), implying no great effect of CaM4 on GSNOR expression under normal conditions. These data suggest that AtCaM4 directly binds to GSNOR and subsequently inhibits its activity, indicating that GSNOR is a specific target of AtCaM4 in the salt signaling pathway.

### CaM4 through GSNOR Regulates NO Production in Salt Resistance

NO bioactivity is controlled by NO synthesis and degradation, which is mainly performed by GSNOR [62,63]. Additionally, the NO system is mainly regulated by the breakdown of GSNO by GSNOR, which is conserved from bacteria to humans [62]. In Arabidopsis, GSNOR, previously known as GSH-dependent formaldehyde dehydrogenase or class III alcohol dehydrogenase (ADH) due to its interaction with primary alcohols, is encoded by a single copy gene, *GSNOR* [35]. GSNOR is an important and widely utilized component of resistance protein signaling networks that controls NO accumulation. However, its role in the salt signaling pathway is not yet clarified.

As shown in Fig 6, *gsnor* mutant plants displayed a higher NO level and enhanced salt tolerance compared to wild-type plants; this phenotype was restored in two complementation lines, and was reversed in two overexpression lines, implying that GSNOR is a negative regulator of salt tolerance according to its inhibition of NO accumulation (Fig 6). We hypothesize that AtCaM4, by binding to and reducing the activity of GSNOR, can enhance the NO level in salt-stressed plants. In the existence of NaCl, NO exhibited positive effects on root growth (Figs 3E and 6F), which should be due to its action on stem cell niche homeostasis through interaction with auxin [64]. However, high levels of NO reduce auxin transport and response by a PIN1-dependent mechanism, and root meristem activity is reduced concomitantly [65], implying that NO through auxin regulates root growth in a concentration-dependent manner.

To further verify the relationship between CaM4-GSNOR and NO in salt signaling, we obtained *GSNOR*-overexpression transgenic lines in a *cam4* background and *cam4gsnor* double mutant plants. Surprisingly, *GSNOR* overexpression reduced both the internal NO level and survival of *cam4* plants, indicating that GSNOR acts downstream of AtCaM4 and inhibits NO accumulation (Fig 7). The deletion of *GSNOR* enhanced the salt tolerance of *cam4* plants accompanied by enhancement of the NO level (Fig 8). One plausible explanation for this is that GSNOR deletion increased the supply of NO in the absence of AtCaM4 so as to affect other different molecular components (i.e. *S*-nitrosylation of specific proteins) to increase salt resistance, suggesting that CaM4-GSNOR stimulates internal NO accumulation under saline conditions.

### The Mechanism Underlying the Effect of AtCaM4 Through GSNOR in Salt-Stressed Plants

When plants are exposed to high concentrations of Na^+^, the excess Na^+^ ions tend to substitute for K^+^ due to physicochemical similarities between Na^+^ and K^+^, leading to plant dysfunction [66]. The ability to control net Na^+^ influx into the cytoplasm and to maintain a minimal Na^+^/K^+^ ratio in the cytoplasm is of great importance in determining plant responses to salinity [67]. NO alleviates salt toxicity in reed [31] and maize [68] through the up-regulation of H^+^-ATPase activity in the plasma membrane and vacuolar membrane, resulting in Na^+^ efflux into the apoplast and vacuole. As a glycophytic species, Arabidopsis is sensitive to moderate levels of NaCl and accumulates a significant amount of Na^+^ when exposed to salinity [19]. NO is associated with salt tolerance in Arabidopsis via attenuation of the NaCl-induced increase in the Na^+^/K^+^ ratio [32].

In the present study, the Na^+^/K^+^ ratio increased with the loss of *AtCaM1* and *AtCaM4* expression under saline conditions, whereas it decreased in the *gsnor* mutant. This situation was enhanced in the *GSNOR*-overexpression lines but partially rescued in the *cam4gsnor* double mutant compared to *cam4* plants, indicating that AtCaM1 and AtCaM4 influence ion absorption through GSNOR (Fig 9).

**Fig 10:**
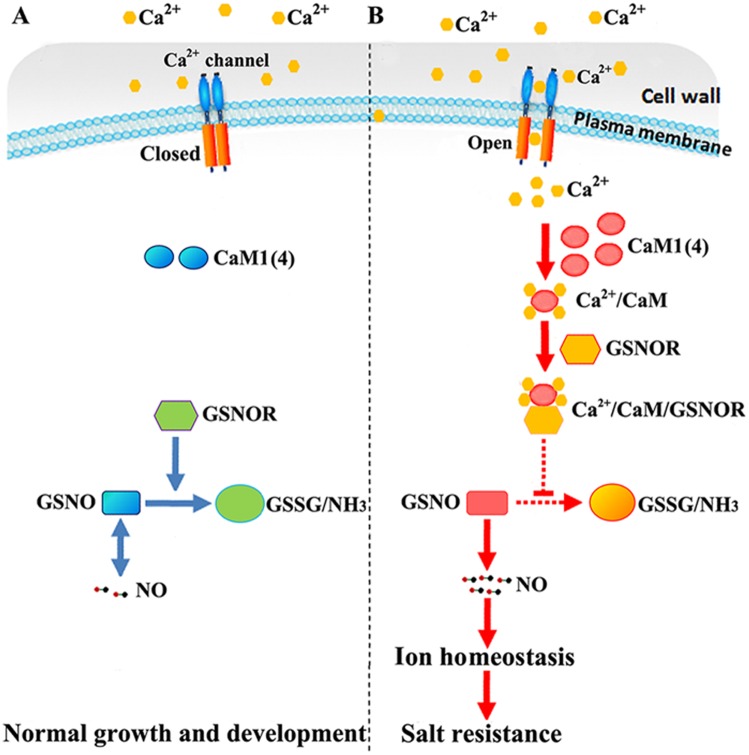
The Ca^2+^/CaM/GSNOR cascade mediates salt resistance in arabidopsis seedlings. (A) Under normal conditions, calcium channel closure limits Ca^2+^ entry so as to prevent CaM binding and Ca^2+^/CaM signaling transduction. The NO level was kept relatively stable by GSNOR. (B) During salt exposure, calcium channels are activated and open. Consequently, the formed Ca^2+^/CaM complex interacts directly with GSNOR and inhibits its activity, thereby stimulating NO accumulation and ion homeostasis to confer salt resistance. Thick arrows indicate normal pathways; dotted arrows show weaker processes; a straight line shows repressive effect. The question mark indicates an incomplete complex.

The present data indicate that AtCaM1 and AtCaM4 regulate ion absorption and affect salt resistance in plants by increasing the cellular level of NO through binding to and inhibiting the activity of GSNOR (Fig 10). Their findings suggest that this plant defensive pathway could share a common evolutionary origin with animals. NO was even reported to control its own generation and scavenging by modulating nitrate assimilation and GSNOR1 activity [69], indicating a feedback inhibition between GSNOR and NO in plants.

## Materials and Methods

### Plant Materials and Growth Conditions

Arabidopsis (*A*. *thaliana* ecotype Columbia) and tobacco (*N*. *tabacum*) were used in this study. The T-DNA insertion line *gsnor* (CS66012) was obtained from the ABRC (http://www.arabidopsis.org/abrc/). The other lines used in this study, *cam2* (SALK_114166), *cam3* (SALK_001357), and *cam4 *(GABI_309E09), were obtained from Drs. Daye Sun and Sujuan Cui (Hebei Normal University). The *cam4gsnor* double mutant was obtained by crossing. RNAi constructs for *AtCaM1* and *AtCaM4* were made and corresponding RNAi plants were produced (the primers used are shown in S1 Table).

Seeds were surface-sterilized in 2% (v/v) sodium hypochlorite for 1 min and then washed thoroughly with water. The sterilized seeds were plated on 0.5× Murashige and Skoog (MS) medium containing 1.5% sucrose and 0.3% agar and kept at 4°C in the dark for 2 days. The plants were then transferred to a growth chamber set at 22°C and 120 μmol/m^2^s on a 16-h day/night cycle.

Tobacco seeds were planted in potting mix (2:1 [v/v] rich soil:vermiculite) and kept in a growth chamber at 23°C with illumination at 120 μmol/m^2^s with a 16-h daily light period. After 3 weeks of growth, the plants were used for transformation.

### Stress Treatments

After germination, seedlings from each line were carefully transferred to a fresh MS agar plate supplemented with 100 mM NaCl. After 7 days of growth on the treatment medium, those seedlings with still green cotyledons were scored as survivors.

For all chemical treatments, 1 ml of 50 μM GSNO, 100 μM cPTIO or 150 μM L-NAME (Sigma-Aldrich, St. Louis, MO) was sprayed onto the leaf surfaces of 5-day-old seedlings after filter-sterilization for 48 h. Control seedlings were treated with water.

### RT-PCR and RT-qPCR

Total RNA (500 ng) was isolated using a PrimeScript RT reagent kit (Takara Bio Inc., Otsu, Japan) for first-stand complementary DNA synthesis. RT-PCR analyses of *AtCaM1* and *AtCaM4* transcription were performed using a Takara RNA PCR (Avian Myeloblastosis Virus) kit version 3.0 (Takara Bio Inc.) with gene-specific primers (S1 Table). RT-qPCR analyses of gene expression were done using an ABI 7500 sequence detection system (Applied Biosystems, Foster City, CA) with SYBR Premix Ex Taq (Takara Bio Inc.) and gene-specific primers (S1 Table). The *18S rRNA* was used as an internal control to normalize all data.

### Fluorescence Microscopy

NO was visualized using the NO-specific fluorescent probe DAF-FM DA (Sigma-Aldrich), according to Wang’s method [70] with some modifications. Seven-day-old wild-type and mutant seedlings were incubated in 1 ml of 0.5× liquid MS medium (pH 5.8) with 10 μM DAF-FM DA for 20 min. Thereafter, the roots were washed three times for 15 min each in 0.5× liquid MS medium prior to visualization using a fluorescence microscope (ELLIPE TE2000-U; Nikon, Tokyo, Japan). The signal intensities were quantified using MetaMorph (Molecular Devices, Sunnyvale, CA).

### Vector Construction and the Generation of Transgenic Plants

To detect the tissue-specific expression of *GSNOR*, an *GSNORpro*:*GUS* construct was generated by introducing the *GSNOR* promoter fragment (2.21 kb) in front of the *GUS* coding sequence in the *Pst*I and *Xba*I sites of pCAMBIA1300. Detailed primer sequences are given in S1 Table. GUS staining assays were performed as described previously [71].

To determine the subcellular localization of GSNOR, *GSNOR* was engineered into pMDC83 with *GFP* at the C-terminus under the control of the 35S promoter. Detailed primer sequences are given in S1 Table. Transiently transfected tobacco leaves were imaged for GFP fluorescence using a Zeiss LSM710 confocal laser scanning microscope (Carl Zeiss AG, Jena, Germany).

For the overexpression of *GSNOR*, *CaM1*, and *CaM4*, the coding sequence of *GSNOR* was introduced into the *Spe*I and *Asc*I sites of PMDC83 under the control of the 35S promoter, while the coding sequences of *CaM1* and *CaM4* were respectively introduced into the *Xba*I and *Bam*HI sites of pCAMBIA1300 under the control of the 35S promoter. To generate constructs for the complementation of *gsnor* and *cam1/4-1*, a genomic DNA fragment of *GSNOR* was amplified and cloned into the *Hind*III and *Asc*I sites of pMDC83, and a genomic DNA fragment of *CaM4* was amplified and cloned into the *Sph*I and *Xba*I sites of pCAMBIA1300. Arabidopsis transformation with *Agrobacterium tumefaciens* (strain GV3101) was performed by the floral dip method [72]. Homozygous T3 transgenic lines were used for further analysis. Detailed primer sequences are given in S1 Table.

### BiFC Assays

BiFC assays were conducted as described previously [73]. Full-length GSNOR and CaM4 were cloned into either pSPYNE-35s or pSPYCE-35s. The resulting constructs were transiently expressed in 3-week-old tobacco leaves by *Agrobacterium*-mediated infiltration (strain GV3101). The YFP fluorescence of the tobacco leaves was imaged 2 days after infiltration using a Zeiss LSM710 confocal laser scanning microscope (Carl Zeiss AG).

### Expression of Recombinant Proteins in *E*. *coli* and Overlay Assays

GST and recombinant GST-GSNOR, GST-GSNOR^N^, GST-GSNOR^C^, CaM4-GST, or CaM4-HiS were expressed in *E*. *coli* strain BL21. Expression of the GST fusion proteins was induced by treatment with 0.1 mM isopropyl 1-thio-β-D-galactopyranoside for 4 h at 30°C; the proteins were purified as described previously [74], with some modifications.

In total, 1 μg of each purified GST fusion protein was separated by 10% SDS-PAGE and transferred to an Immobilon-P (polyvinylidene difluoride) membrane (Merck KGaA, Darmstadt, Germany), after which the expressed GST fusion proteins were detected using a monoclonal GST-specific antibody. To examine the CaM4 binding ability of the recombinant proteins, a duplicate blot was probed with 1 μg of CaM4-His (carrying a 6× histidine tag at the C-terminus) in the presence of 0.1 mM CaCl_2_ or 0.1 EGTA in 10 ml of TBST and then probed with an anti-His monoclonal antibody (Proteintech Group Inc., Chicago, IL). Bound CaM4 was visualized using an ECL detection system (GE Healthcare, Wauwatosa, WI).

### Protein Extraction, Immunoblotting, and *In Vivo* Co-IP

Arabidopsis proteins were extracted from 10-day-old seedlings using NEB buffer (20 mM HEPES, pH 7.5, containing 40 mM KCl, 1 mM EDTA, 1 mM PMSF, and 1× protease inhibitor cocktail [Roche, Basel, Switzerland]) after centrifugation at 12,000 × *g*, 4°C, for 20 min. Co-IP was performed as described previously [75,76], with minor modifications. In total, 50 μl of the supernatant was collected as input. The rest of the supernatant was used for immunoprecipitation using 10 μl of GFP-Trap agarose beads (ChromoTek, Martinsried, Germany). After incubation for 2 h at 4°C, the beads were washed five times in wash buffer (20 mM HEPES, pH 7.5, 40 mM KCl, and 0.1% Triton X-100). An appropriate amount of 2× SDS sample buffer was then added to the beads, which were boiled for 10 min at 100°C, and then subjected to 10% SDS-PAGE for immunoblot analysis using antibodies against GFP (G1544-100UG; Sigma-Aldrich) and GSNOR (AS09647; Agrisera, Vännäs, Sweden). All experiments were repeated independently three times; representative results from a single experiment are shown.

### Measurement of GSNOR Activity

Total protein was extracted from the leaves of 7-d-old seedlings using NEB buffer (20 mM HEPES, pH 7.5, 40 mM KCl, and 1 mM EDTA) by centrifugation at 20,000g, 4°C for 20 min.

A total of each supernatant was incubated with 20 μl protein A beads (GE Healthcare) bound to GSNOR antibodies After 2 h, the beads were washed five times in wash buffer (20 mM HEPES, pH 7.5, 40 mM KCl, and 0.1% Triton X-100). An appropriate amount of 0.1M citric acid PH 3.0 was then added to elute the antibody. Immediately the eluted fractions were neutralize with 1M Tris-HCl, PH8.5-PH7.4 to obtain purified GSNOR protein.

GSNOR activity was measured by monitoring the decomposition of NADH [77,78]. The oxidation of NADH, dependent on the presence of the substrate GSNO, was determined spectrophotometrically at 340 nm. A crude leaf (25 mg) extract of Arabidopsis seedlings was prepared in 100 μl of 0.05 M HEPES buffer (20% glycerol, 10 mM MgCl_2_, 1 mM EDTA, 1 mM benzamidine, and 1 mM Ɛ-aminocaproic acid, pH 8.0), centrifuged to remove insoluble material, and then clarified with a desalting column (Zeba desalting column; Pierce, Rockford, IL). Enzyme activity was determined at 25°C by incubating the desalted fraction (10 μl) in 180 μl of 0.1 M phosphate buffer containing 10 μl of 6 mM NADH as a cofactor and 10 ml of 6 mM GSNO as the substrate. GSNOR activity was monitored for 1 min after the addition of NADH using an Agilent 8453 UV spectrophotometer (Agilent Technologies, Santa Clara, CA). The rates were corrected for background NADH decomposition in each extract containing no GSNO. The rates were averaged over selected intervals during which the decrease in absorbance was linear. The final NADH decomposition values were normalized against the amount of total protein. All data given are the means of three independent experiments.

### Determination of SNO Content

Total SNO levels were determined by Saville’s method [48,79]. Proteins were extracted in 100 mM Tris HCl, pH 6.8. The extracts were incubated for 5 min with an equivalent volume of solution A (1% sulfanilamide dissolved in 0.5 M HCl) in the presence or absence of solution B (solution A plus 0.2% HgCl_2_), allowing the development of the diazonium salt. The formation of the azo dye product was obtained by reacting the two samples for an additional 5 min with an equal volume of solution C [0.02% of *N*-(1-naphthyl) ethylenediamine dihydrochloride dissolved in 0.5 M HCl], and the absorbance was subsequently read at 550 nm with a spectrophotometer. *S*-NOHCy was quantified as the difference of absorbance between solution B and A (B–A), comparing the values with a standard curve made from a solution of GSNO (Sigma-Aldrich). Low *M*r SNOs were determined in the fraction passing through a 5 K cut of ultrafiltration membrane. The results were normalized against whole cell-lysate protein content.

### Determination of Elemental Concentrations

To determine the Na^+^ and K^+^ contents in plant tissue, samples were harvested, oven-dried for at least 24 h at 80°C, weighed, and then digested in concentrated (69%, v/v) HNO_3_ for at least 12 h for elemental extraction. The concentrations of Na^+^ and K^+^ were determined in appropriately diluted samples in an air-acetylene flame by atomic absorption spectrophotometry using a double-beam optical system with deuterium arc background correction (AAnalyst 100; PerkinElmer, Waltham, MA). Measurement of the Na^+^ and K^+^ concentrations was performed as described previously [80].

### Accession Numbers

The sequence data from this article can be found in the Arabidopsis Genome Initiative or GenBank/EMBL database under the following accession numbers: At5g43940 for *GSNOR*, At5g37780 for *AtCaM1*, At2g27030 for *AtCaM2*, At3g56800 for *AtCaM3*, At1g66410 for *AtCaM4*, At2g41110 for *AtCaM5*, At5g21274 for *AtCaM6*, and At3g43810 for *AtCaM7*.

## Supporting Information

S1 Fig Wild-type, *cam1-1*, *cam1-2*, *cam4*, *cam1/4-1*, and *cam1/4-2* plants grown under normal conditions. (A) The construction of *amiCaM1*. The specific base sites used to construct the artificial microRNA vector are shown in blue. (B) Phenotypic comparison of 4-week-old wild-type, *cam1*, and *cam4* plants grown under normal conditions.
(PDF)

S2 Fig The Loss of *AtCaM1* and *AtCaM4* has no effect on other *AtCaMs*. RT-qRCR analysis of *AtCaM2* (A), *AtCaM3* (B), *AtCaM5* (C), *AtCaM6* (D), and *AtCaM7* (E) transcription in wild-type, *cam1-*1, *cam1-2*, *cam4*, *cam1/4-1*, and *cam1/4-2* plants. The experiments were repeated three times with similar results. Each data point represents the mean ± SD (n = 3). Asterisks indicate a significant difference relative to Col (Student’s *t*-test, *P < 0.05).
(PDF)

S3 Fig The phenotype of the *amiCaM1/4* lines under normal growth conditions. (A) The construction of *amiCaM1/4*. The specific base sites used to construct the artificial microRNA vector are shown in blue. (B) Phenotypic comparison of 4-week-old wild-type, *cam1/4-3*, and *cam1/4-4* plants under normal growth conditions. (C, D) RT-qPCR analysis of the *AtCaM1* (C) and *AtCaM4* (D) transcript levels in wild-type, *cam1/4-3*, and *cam1/4-4* plants. *ACTIN2* was used as an internal control. The experiments were repeated three times with similar results. Each data point represents the mean ± SD (n = 3). Asterisks indicate a significant difference relative to Col (Student’s *t*-test, *P < 0.05, **P < 0.01, and ***P < 0.001).
(PDF)

S4 Fig Salt sensitivity analysis of *cam1/4-3* and *cam1/4-4* seedlings. (A) Salt stress sensitivity of 7-day-old wild-type, *cam1/4-3*, and *cam1/4-4* seedlings in 0.5× MS medium with or without 100 mM NaCl. The experiments were repeated three times with similar results. (B) Survival ratios of the seedlings after salt treatment. Each data point represents the mean ± SE (n = 30). Asterisks indicate a significant difference relative to Col (Student’s *t*-test, *P < 0.05 and **P < 0.01).
(PDF)

S5 Fig Salt sensitivity analysis of *cam2* and *cam3* seedlings. (A) Salt stress sensitivity of 7-day-old wild-type, *cam2*, and *cam3* seedlings in 0.5× MS medium with or without 100 mM NaCl. The experiments were repeated three times with similar results. (B) Survival ratios of the seedlings after salt treatment. Each data point represents the mean ± SE (n = 30).
(PDF)

S6 Fig Phenotype of the *AtCaM4* complementation lines under normal growth conditions. (A) RT-qPCR analysis of *AtCaM1* and *AtCaM4* transcription in wild-type and *cam1/4-1* plants and in two *AtCaM4* complementation lines (4COM1 and 4COM2). *ACTIN2* was used as an internal control. The experiments were repeated three times with similar results. Each data point represents the mean ± SD (n = 3). Asterisks indicate a significant difference relative to Col (Student’s *t*-test, **P < 0.01 and ***P < 0.001). (B) Morphological phenotype of 4-week-old plants under normal growth conditions.
(PDF)

S7 Fig Salt sensitivity analysis of the *AtCaM4* complementation lines. (A) Salt stress sensitivity of 7-day-old wild-type plants, *cam1/4-1* mutant plants, and two *AtCaM4* complementation lines at the seedling stage in 0.5× MS medium with or without 100 mM NaCl. The experiments were repeated three times with similar results. (B) Survival ratios of the seedlings after salt treatment. Each data point represents the mean ± SE (n = 30). Asterisks indicate a significant difference relative to Col (Student’s *t*-test, **P < 0.01).
(PDF)

S8 Fig Phenotypes of the *AtCaM1* overexpression lines under normal and high-salt conditions. (A) RT-PCR analysis of *AtCaM1* transcription in wild-type, 1OE1 and 1OE2 plants. *ACTIN2* was used as an internal control. (B) Phenotypic comparison of 4-week-old plants under normal conditions. (C) Phenotypic comparison of 7-day-old seedlings in 0.5× MS medium with or without 125 mM NaCl. The experiments were repeated three times with similar results. (D) Survival ratios of the seedlings after salt treatment. Each data point represents the mean ± SE (n = 30).
(PDF)

S9 Fig Phenotypes of the *AtCaM4* overexpression lines under normal and high-salt conditions. (A) RT-PCR analysis of *AtCaM4* transcription in wild-type, 4OE1 and 4OE2 plants. *ACTIN2* was used as an internal control. (B) Phenotypic comparison of 4-week-old plants under normal conditions. (C) Phenotypic comparison of 7-day-old seedlings in 0.5× MS medium with or without 125 mM NaCl. The experiments were repeated three times with similar results. (D) Survival ratios of the seedlings after salt treatment. Each data point represents the mean ± SE (n = 30).
(PDF)

S10 Fig The effect of cPTIO on the DAF-FM fluorescence density in the wild-type seedlings under high-salt conditions. (A) The fluorescence density in the roots of 5-day-old wild-type seedlings grown in 0.5× MS liquid medium containing 100 mM NaCl supplemented with 0, 0.3, 0.4, 0.5, or 1.0 mM cPTIO for the next 2 days was detected by DAF-FM DA staining. The experiments were repeated three times with similar results. Bar = 50 μm. (B) Relative DCF fluorescence densities in the roots. Each data point represents the mean ± SE (n = 20). Asterisks indicate a significant difference relative to Col (Student’s *t*-test, *P < 0.05 and **P < 0.01).
(PDF)

S11 Fig The structure of AtCaM4 bound to the GSNOR peptide. The AtCaM4 peptide backbone, Ca^2+^ ions, and GSNOR peptide backbone are shown in blue, pink, and green, respectively. The structures were visualized using WebLab ViewerLite (Accelrys).
(PDF)

S12 Fig The effect of AtCaM4 on GSNOR activity *in vitro*. (A) Coomassie blue-stained recombinant AtCaM4 and GSNOR (indicated by arrows). (B) Relative *in vitro* GSNOR activity in the presence of different CaM4-His/GST-GSNOR ratios. The experiments were repeated three times with similar results. Each data point represents the mean ± SD (n = 3).
(PDF)

S13 Fig The *GSNOR* expression pattern. (A) RT-qPCR analysis of *GSNOR* transcription in 7-day-old wild-type seedlings kept in 0.5× MS liquid medium with or without 50 mM NaCl for 0–12 h. *ACTIN2* was used as an internal control. The experiments were repeated three times with similar results. Each data point represents the mean ± SD (n = 3). Asterisks indicate a significant difference relative to 0 h (Student’s *t*-test, *P < 0.05). (B) Tissue-specific expression of *GSNOR* in 10-day-old Arabidopsis seedlings. (C) Subcellular localization of the GSNOR-GFP fusion protein in tobacco epidermal cells.
(PDF)

S14 Fig Subcellular localization of the CaM4-GFP fusion protein in tobacco epidermal cells. (PDF)

S1 Table Primers sequence used in this study. (PDF)
