# Supplementary material for: Arabidopsis CaM1 and CaM4 Promote Nitric Oxide Production and Salt Resistance by Inhibiting S-Nitrosoglutathione Reductase via Direct Binding
Source: PLoS Genet. 2016 Sep 29;12(9):e1006255. doi: 10.1371/journal.pgen.1006255 (PMC5042403; doi:10.1371/journal.pgen.1006255)
Supplement: S1 Fig — (A) The construction of amiCaM1. The specific base sites used to construct the artificial microRNA vector are shown in blue. (B) Phenotypic comparison of 4-week-old wild-type, cam1, and cam4 plants grown under normal conditions. (PDF) [file pgen.1006255.s001.pdf]

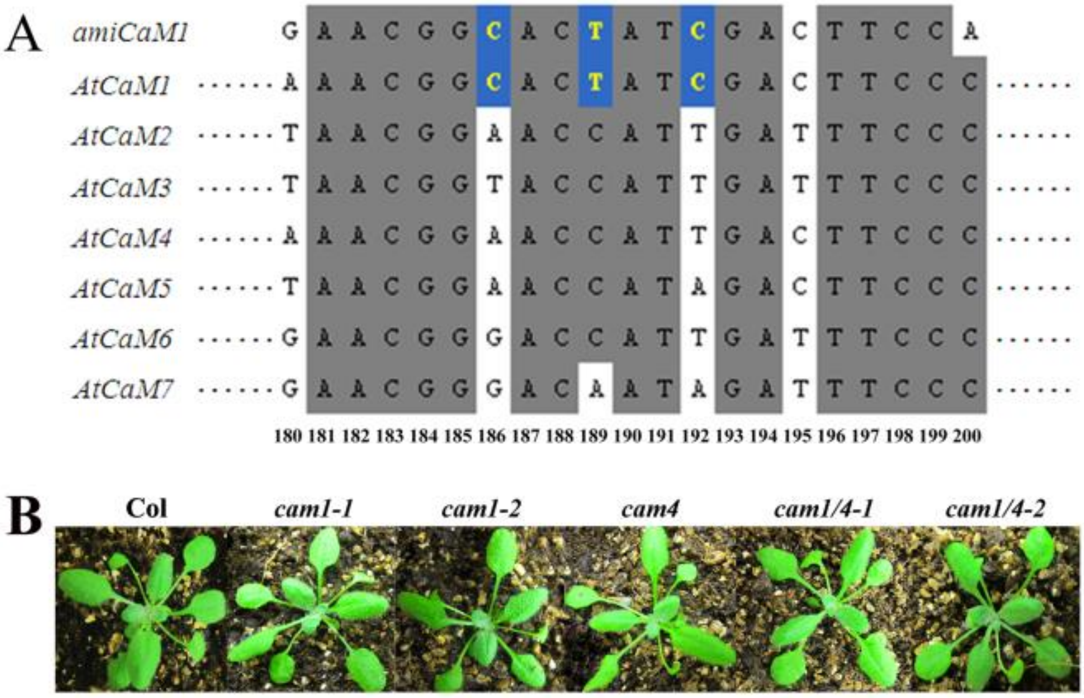
A
amiCaM1 G A A C G G C A C T A T C G A C T T C C A
AtCaM1 ······A A A C G G C A C T A T C G A C T T C C C······
AtCaM2 ······T A A C G G A A C C A T T G A T T T C C C······
AtCaM3 ······T A A C G G T A C C A T T G A T T T C C C······
AtCaM4 ······A A A C G G A A C C A T T G A C T T C C C······
AtCaM5 ······T A A C G G A A C C A T A G A C T T C C C······
AtCaM6 ······G A A C G G G A C C A T T G A T T T C C C······
AtCaM7 ······G A A C G G G A C A A T A G A T T T C C C······
180 181 182 183 184 185 186 187 188 189 190 191 192 193 194 195 196 197 198 199 200
B
Col
cam1-1
cam1-2
cam4
cam1/4-1
cam1/4-2
