# Supplementary figures and images for: Arabidopsis CaM1 and CaM4 Promote Nitric Oxide Production and Salt Resistance by Inhibiting S-Nitrosoglutathione Reductase via Direct Binding
Source: PLoS Genet. 2016 Sep 29;12(9):e1006255. doi: 10.1371/journal.pgen.1006255 (PMC5042403; doi:10.1371/journal.pgen.1006255)

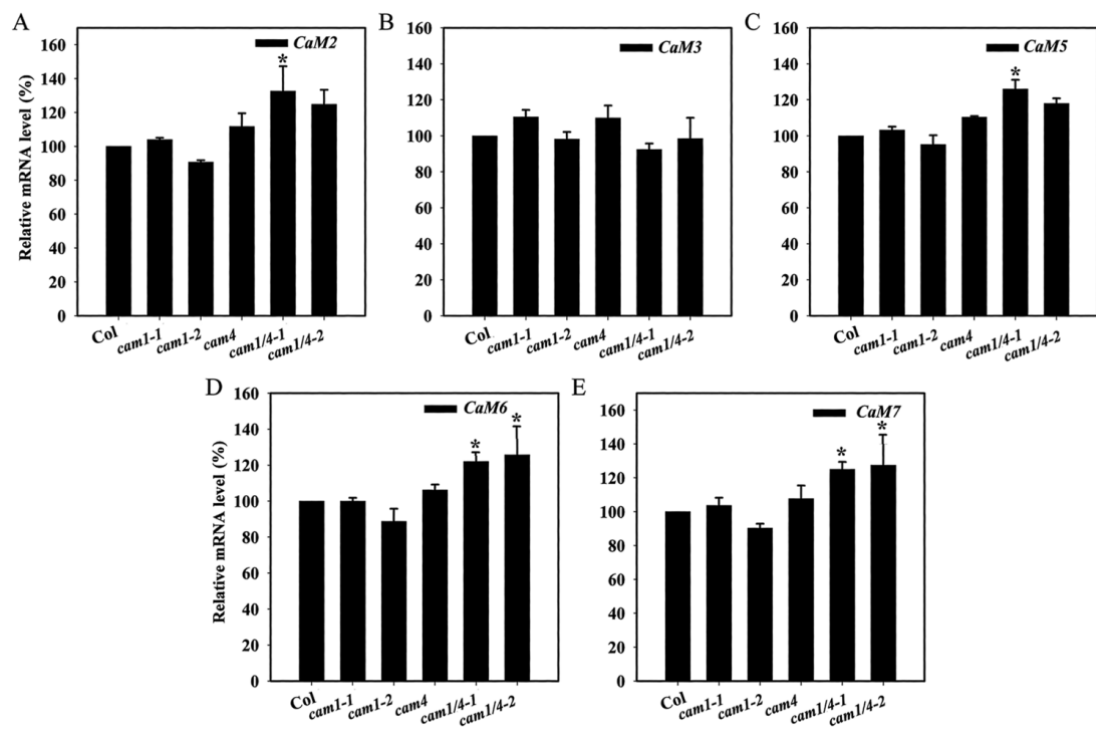

Supplement: S2 Fig — RT-qRCR analysis of AtCaM2 (A), AtCaM3 (B), AtCaM5 (C), AtCaM6 (D), and AtCaM7 (E) transcription in wild-type, cam1-1, cam1-2, cam4, cam1/4-1, and cam1/4-2 plants. The experiments were repeated three times with similar results. Each data point represents the mean ± SD (n = 3). Asterisks indicate a significant difference relative to Col (Student’s t-test, *P < 0.05). (PDF) [file pgen.1006255.s002.pdf]

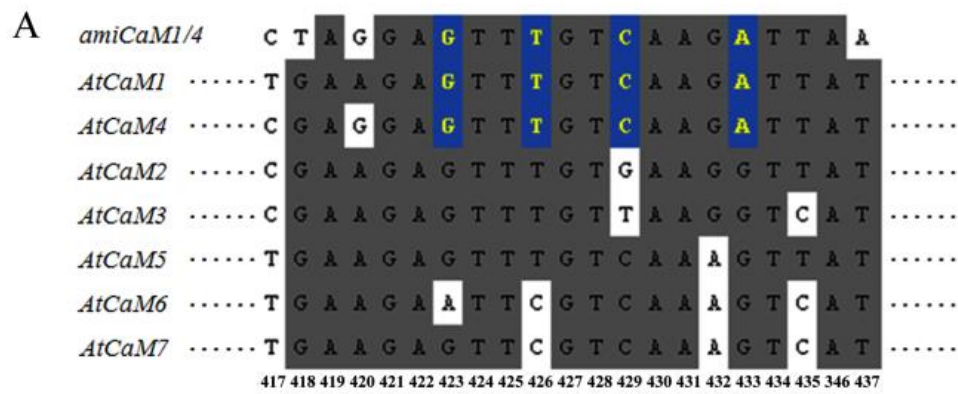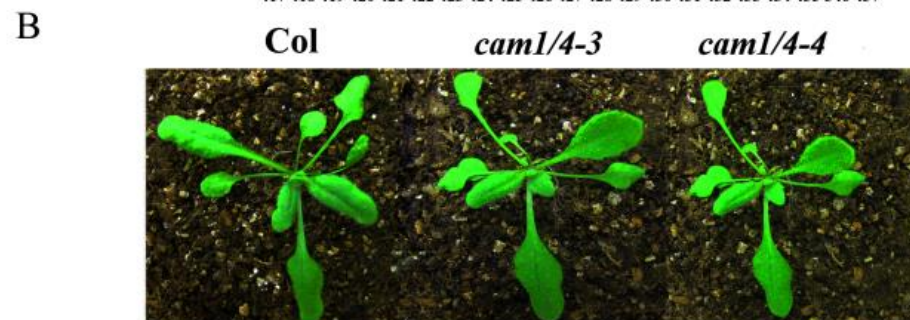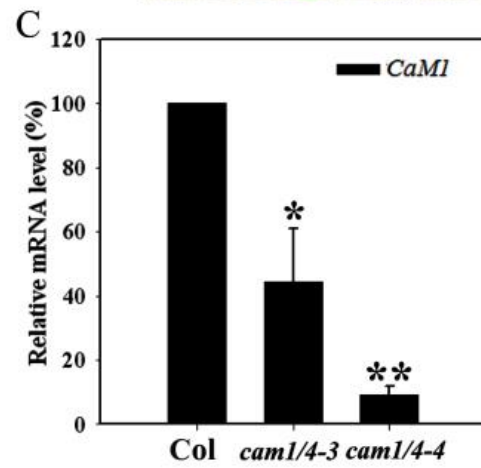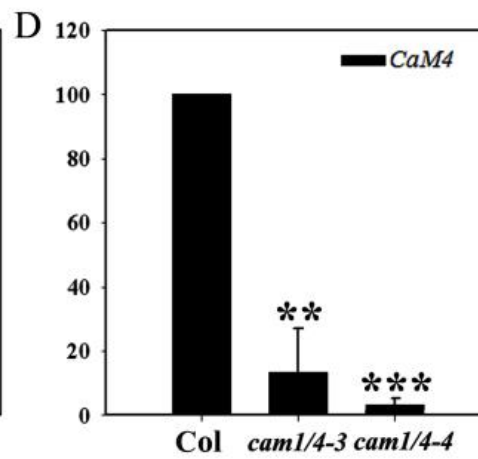

Supplement: S3 Fig — (A) The construction of amiCaM1/4. The specific base sites used to construct the artificial microRNA vector are shown in blue. (B) Phenotypic comparison of 4-week-old wild-type, cam1/4-3, and cam1/4-4 plants under normal growth conditions. (C, D) RT-qPCR analysis of the AtCaM1 (C) and AtCaM4 (D) transcript levels in wild-type, cam1/4-3, and cam1/4-4 plants. ACTIN2 was used as an internal control. The experiments were repeated three times with similar results. Each data point represents the mean ± SD (n = 3). Asterisks indicate a significant difference relative to Col (Student’s t-test, *P < 0.05, **P < 0.01, and ***P < 0.001). (PDF) [file pgen.1006255.s003.pdf]

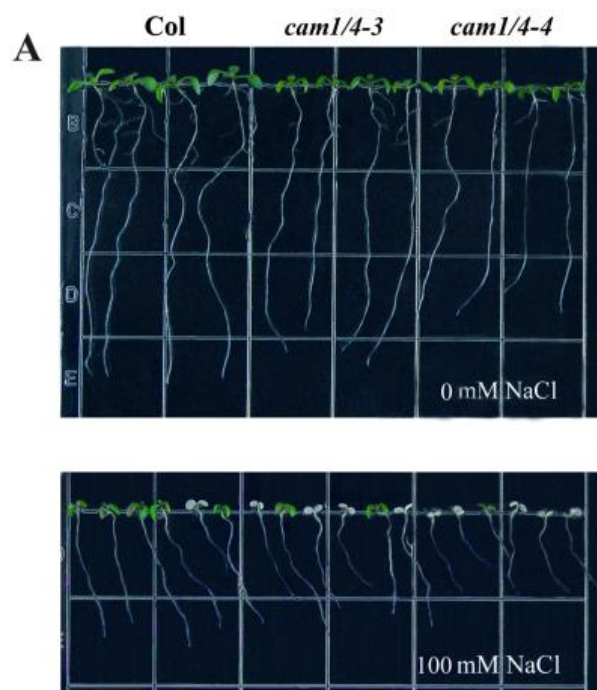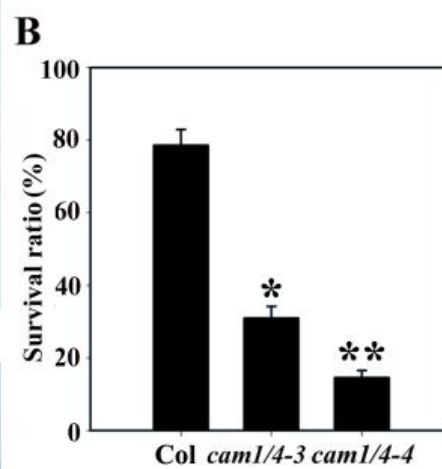

Supplement: S4 Fig — (A) Salt stress sensitivity of 7-day-old wild-type, cam1/4-3, and cam1/4-4 seedlings in 0.5× MS medium with or without 100 mM NaCl. The experiments were repeated three times with similar results. (B) Survival ratios of the seedlings after salt treatment. Each data point represents the mean ± SE (n = 30). Asterisks indicate a significant difference relative to Col (Student’s t-test, *P < 0.05 and **P < 0.01). (PDF) [file pgen.1006255.s004.pdf]

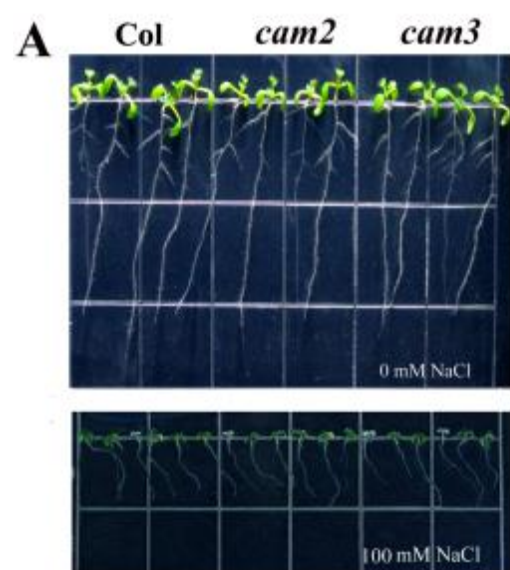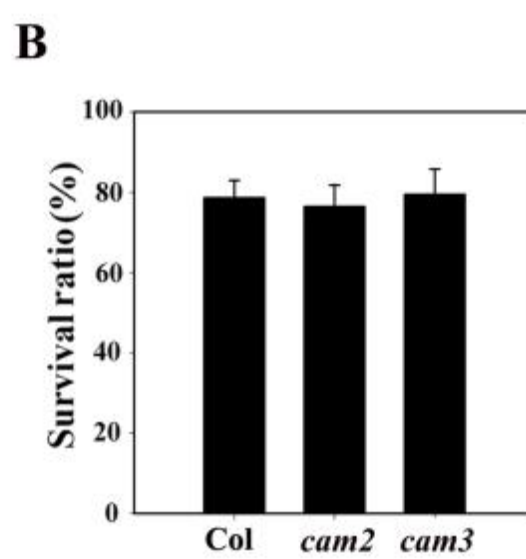

Supplement: S5 Fig — (A) Salt stress sensitivity of 7-day-old wild-type, cam2, and cam3 seedlings in 0.5× MS medium with or without 100 mM NaCl. The experiments were repeated three times with similar results. (B) Survival ratios of the seedlings after salt treatment. Each data point represents the mean ± SE (n = 30). (PDF) [file pgen.1006255.s005.pdf]

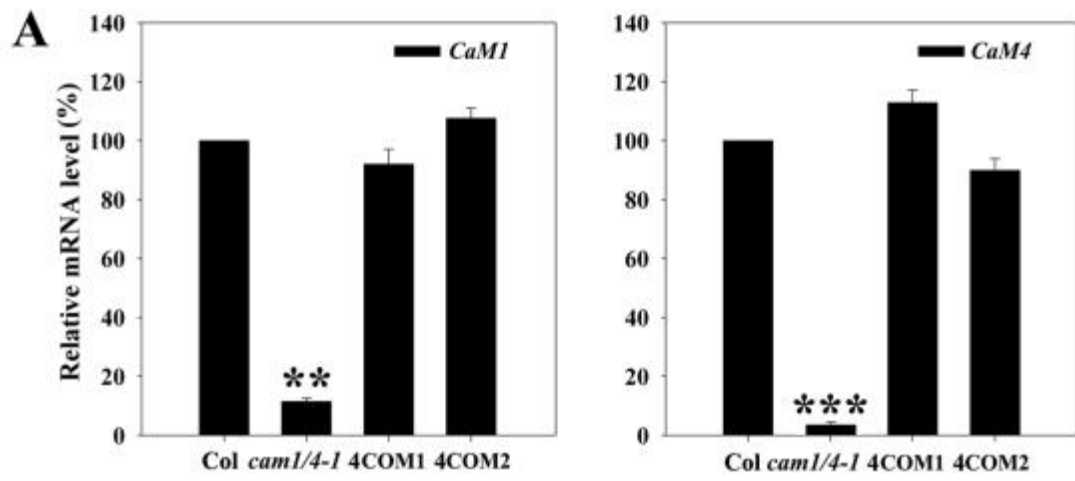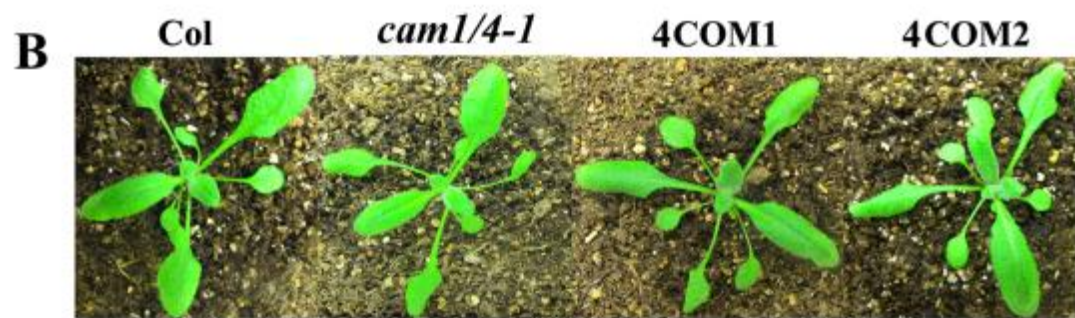

Supplement: S6 Fig — (A) RT-qPCR analysis of AtCaM1 and AtCaM4 transcription in wild-type and cam1/4-1 plants and in two AtCaM4 complementation lines (4COM1 and 4COM2). ACTIN2 was used as an internal control. The experiments were repeated three times with similar results. Each data point represents the mean ± SD (n = 3). Asterisks indicate a significant difference relative to Col (Student’s t-test, **P < 0.01 and ***P < 0.001). (B) Morphological phenotype of 4-week-old plants under normal growth conditions. (PDF) [file pgen.1006255.s006.pdf]

A    Col    *cam1/4-1*    4COM1    4COM2

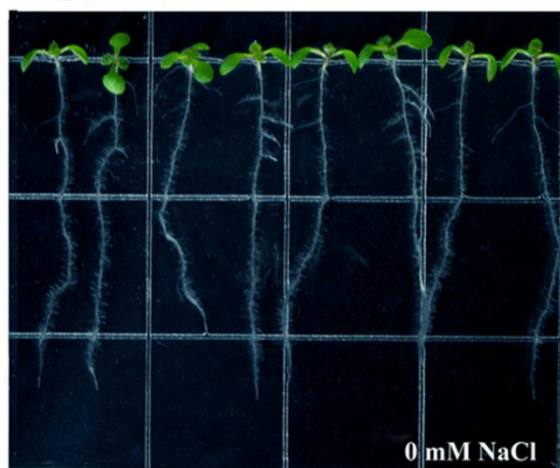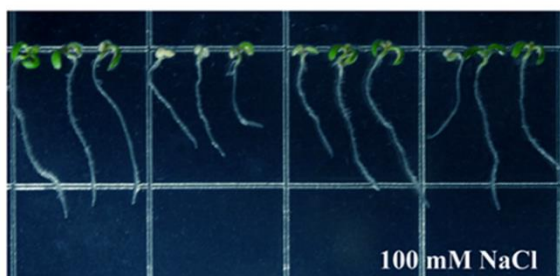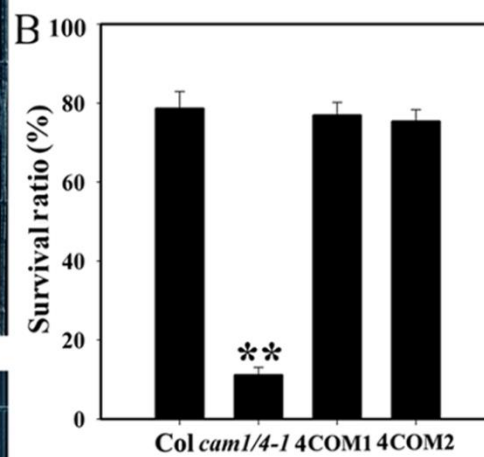

Supplement: S7 Fig — (A) Salt stress sensitivity of 7-day-old wild-type plants, cam1/4-1 mutant plants, and two AtCaM4 complementation lines at the seedling stage in 0.5× MS medium with or without 100 mM NaCl. The experiments were repeated three times with similar results. (B) Survival ratios of the seedlings after salt treatment. Each data point represents the mean ± SE (n = 30). Asterisks indicate a significant difference relative to Col (Student’s t-test, **P < 0.01). (PDF) [file pgen.1006255.s007.pdf]

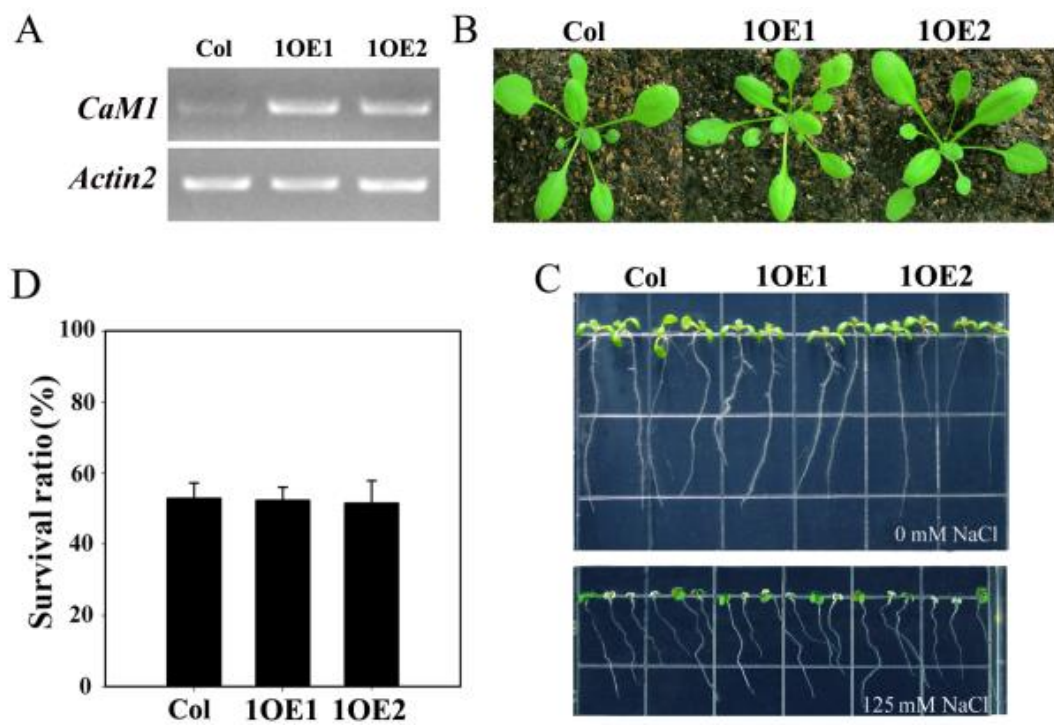

Supplement: S8 Fig — (A) RT-PCR analysis of AtCaM1 transcription in wild-type, 1OE1 and 1OE2 plants. ACTIN2 was used as an internal control. (B) Phenotypic comparison of 4-week-old plants under normal conditions. (C) Phenotypic comparison of 7-day-old seedlings in 0.5× MS medium with or without 125 mM NaCl. The experiments were repeated three times with similar results. (D) Survival ratios of the seedlings after salt treatment. Each data point represents the mean ± SE (n = 30). (PDF) [file pgen.1006255.s008.pdf]

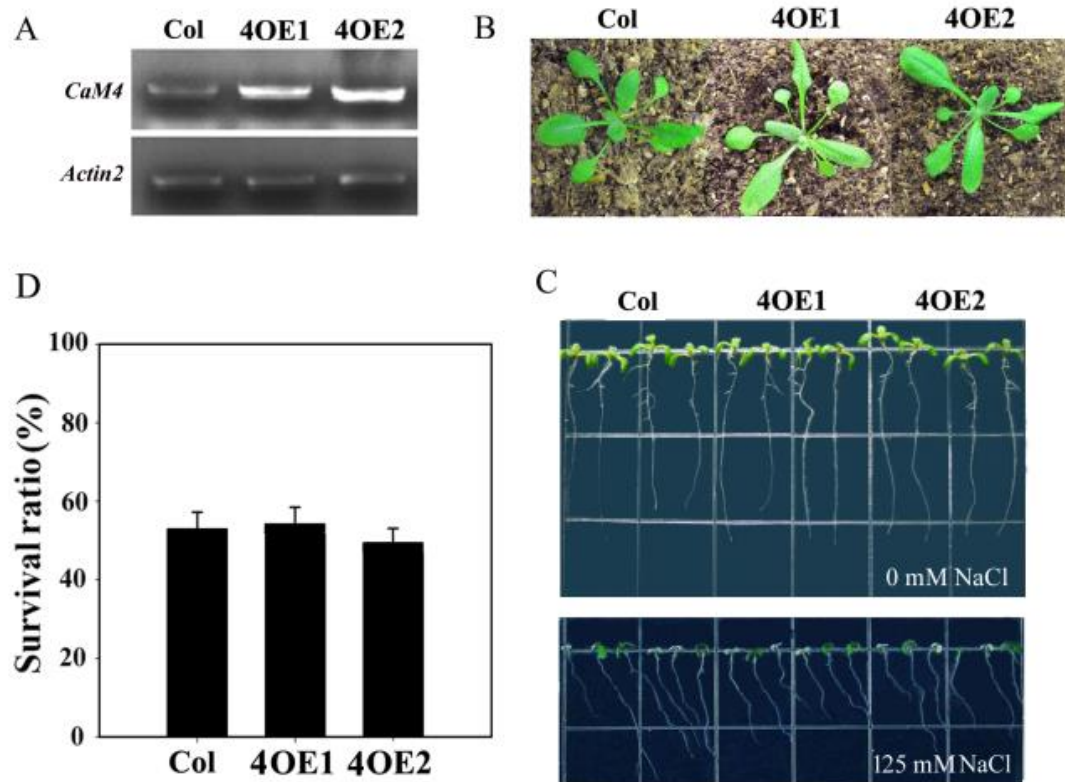

Supplement: S9 Fig — (A) RT-PCR analysis of AtCaM4 transcription in wild-type, 4OE1 and 4OE2 plants. ACTIN2 was used as an internal control. (B) Phenotypic comparison of 4-week-old plants under normal conditions. (C) Phenotypic comparison of 7-day-old seedlings in 0.5× MS medium with or without 125 mM NaCl. The experiments were repeated three times with similar results. (D) Survival ratios of the seedlings after salt treatment. Each data point represents the mean ± SE (n = 30). (PDF) [file pgen.1006255.s009.pdf]

**A**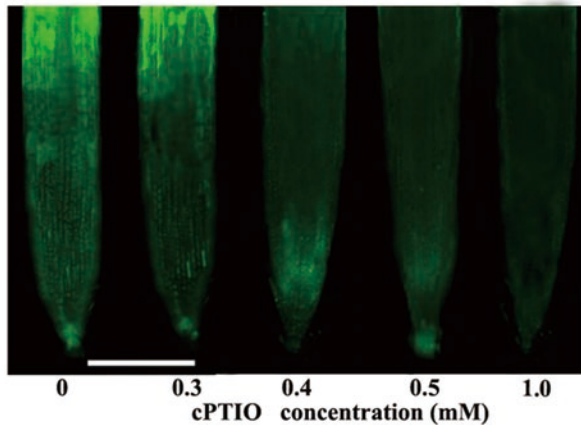**B**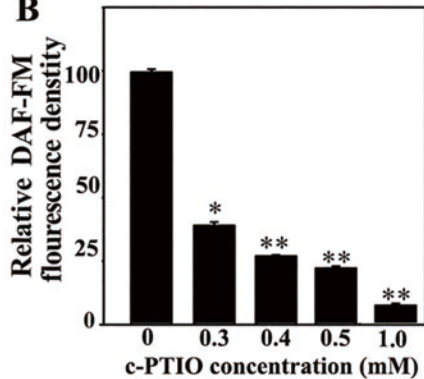

Supplement: S10 Fig — (A) The fluorescence density in the roots of 5-day-old wild-type seedlings grown in 0.5× MS liquid medium containing 100 mM NaCl supplemented with 0, 0.3, 0.4, 0.5, or 1.0 mM cPTIO for the next 2 days was detected by DAF-FM DA staining. The experiments were repeated three times with similar results. Bar = 50 μm. (B) Relative DCF fluorescence densities in the roots. Each data point represents the mean ± SE (n = 20). Asterisks indicate a significant difference relative to Col (Student’s t-test, *P < 0.05 and **P < 0.01). (PDF) [file pgen.1006255.s010.pdf]

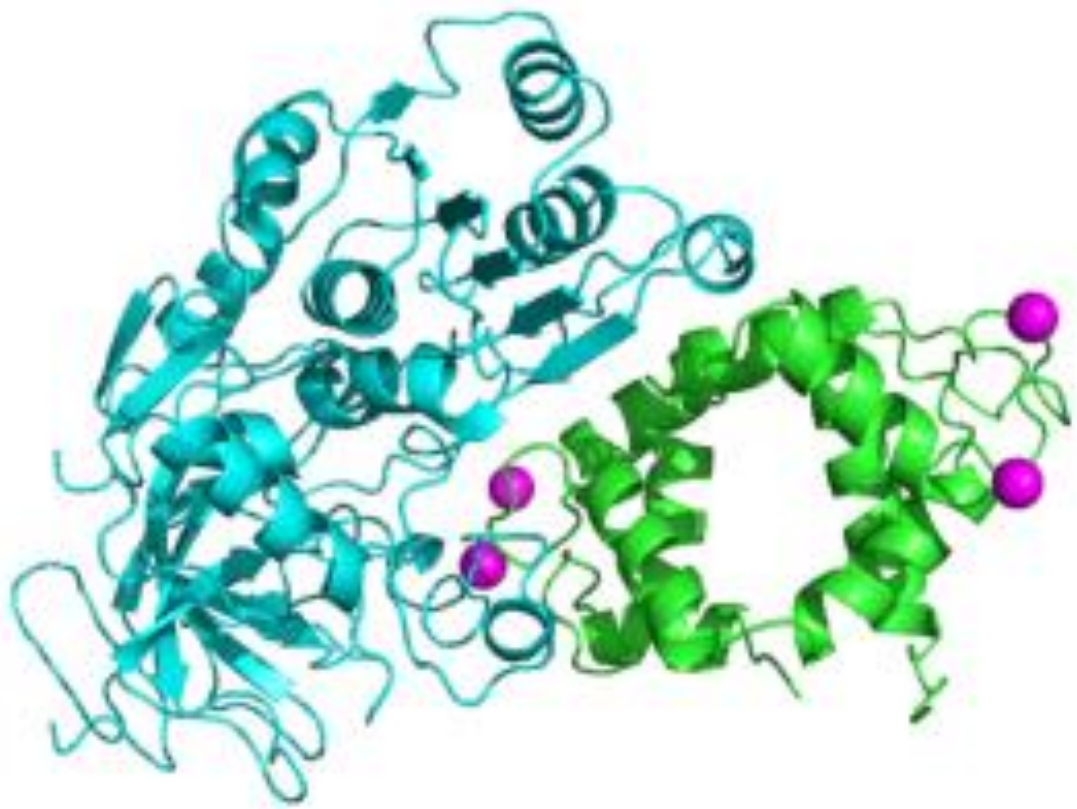

Supplement: S11 Fig — The AtCaM4 peptide backbone, Ca2+ ions, and GSNOR peptide backbone are shown in blue, pink, and green, respectively. The structures were visualized using WebLab ViewerLite (Accelrys). (PDF) [file pgen.1006255.s011.pdf]

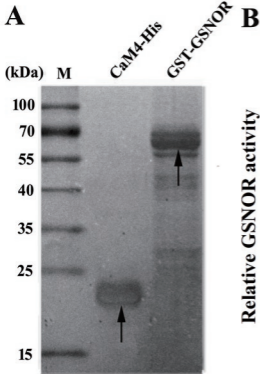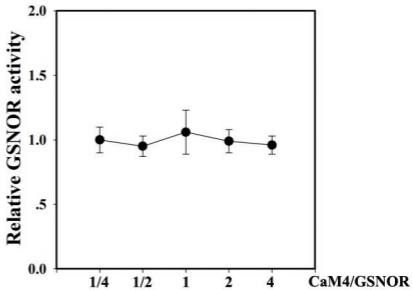

Supplement: S12 Fig — (A) Coomassie blue-stained recombinant AtCaM4 and GSNOR (indicated by arrows). (B) Relative in vitro GSNOR activity in the presence of different CaM4-His/GST-GSNOR ratios. The experiments were repeated three times with similar results. Each data point represents the mean ± SD (n = 3). (PDF) [file pgen.1006255.s012.pdf]

**A**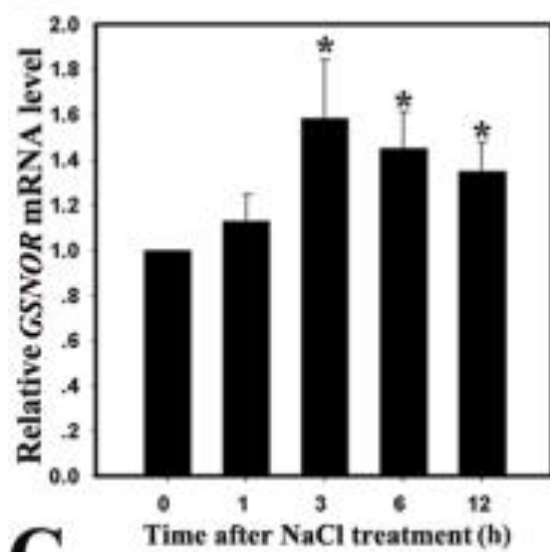**B**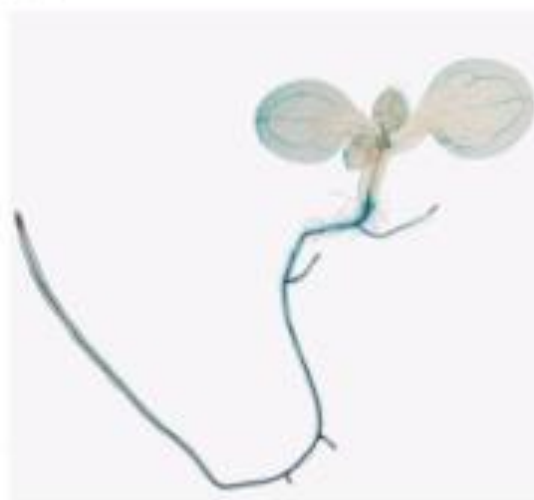**C**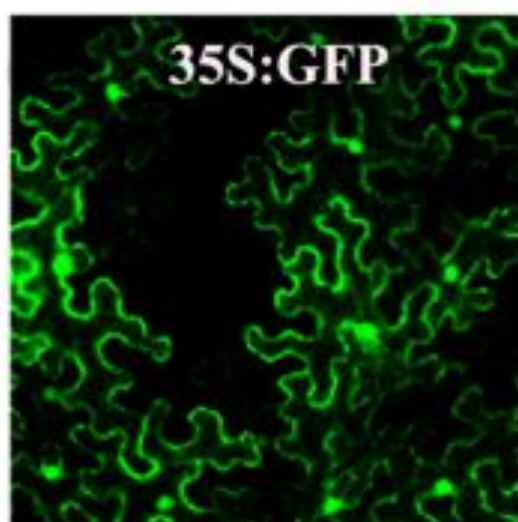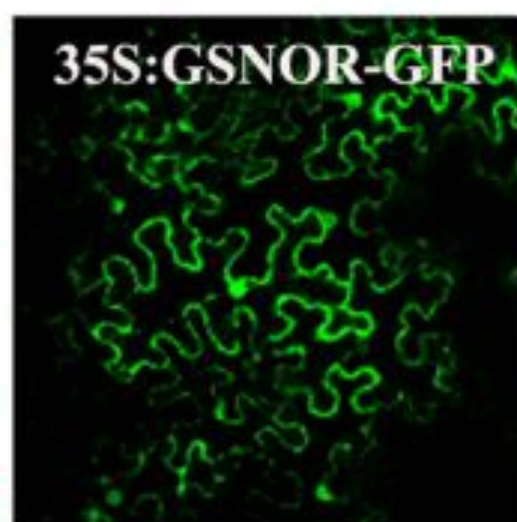

Supplement: S13 Fig — (A) RT-qPCR analysis of GSNOR transcription in 7-day-old wild-type seedlings kept in 0.5× MS liquid medium with or without 50 mM NaCl for 0–12 h. ACTIN2 was used as an internal control. The experiments were repeated three times with similar results. Each data point represents the mean ± SD (n = 3). Asterisks indicate a significant difference relative to 0 h (Student’s t-test, *P < 0.05). (B) Tissue-specific expression of GSNOR in 10-day-old Arabidopsis seedlings. (C) Subcellular localization of the GSNOR-GFP fusion protein in tobacco epidermal cells. (PDF) [file pgen.1006255.s013.pdf]

**35S:GFP**

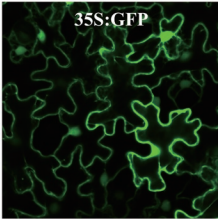

**35S:CaM4-GFP**

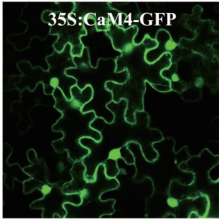

Supplement: S14 Fig — (PDF) [file pgen.1006255.s014.pdf]
