# Supplementary material for: Arabidopsis CaM1 and CaM4 Promote Nitric Oxide Production and Salt Resistance by Inhibiting S-Nitrosoglutathione Reductase via Direct Binding
Source: PLoS Genet. 2016 Sep 29;12(9):e1006255. doi: 10.1371/journal.pgen.1006255 (PMC5042403; doi:10.1371/journal.pgen.1006255)
Supplement: S1 Table — (PDF) [file pgen.1006255.s015.pdf]

S1 Table. Primers Sequence Used in This Study.

Purpose	primer name	sequence (5'-3')
CaM1 construction	CaM1-Xba1-F	GCTCTAGAAAGGAAGAGAAGAAAGACGAT
	CaM1-BamH1-R	CGGGATCCCTTAGCCATCATAATCTTGAC
CaM4 construction	CaM4-Xba1-F	GCTCTAGAAACCAAAGAAACGAGAAGAAG
	CaM4-BamH1-R	CGGGATCCCTTAGCCATCATAATCTTGACA
Cloning of *CaM1* promoter	CaM1Pro-F	AACTGCAGGTGACCACGTATTTGAAATTG
	CaM1Pro-R	GCTCTAGATGTCTTTACTTAATGAGTTTT
Cloning of *CaM4* promoter	CaM4Pro-F	ACATGCATGCCATCAGCCTCCAACAACTC
	CaM4Pro-R	GCTCTAGATTTCGTTTCAGAGAAGAAATGAT
Cloning of *GSNOR* promoter	GSNORGUS-F	AACTGCAGCGATAATTCGGTAAGCACGAGAC
	GSNORGUS-R	GCTCTAGATGACGCAGGAAGAAATGAGAGAG
Overexpression of *GSNOR*	GSNORGFP-F	GGACTAGTATGGCGACTCAAGGTCAGGTTAT
	GSNORGFP-R	TTGGCGCGCCCTTTGCTGGTATCGAGGACACAAC
Complementation of *gsnor*	GSNOR-F	CCCAAGCTTCATTCCTGCCGAATTGATGCG
	GSNOR-R	TTGGCGCGCCCTTTGCTGGTATCGAGGACACAAC
Co-IP assay	CaM4P-F-CACC	CACCCATCAGCCTCCAACAACTC
	CaM4-R	CTTAGCCATCATAATCTTGACA
Bifc assay	CaM4-F-CACC	CACCATGGCGGATCAGCTAACTGA
	CaM4-R	CTTAGCCATCATAATCTTGACA
	GSNOR-F-CACC	CACCATGGCGACTCAAGGTCAGG
	GSNOR-R	TTTGCTGGTATCGAGGACACAAC
Overlay	GSNOR-N-F	CGGAATTCATGGCGACTCAAGGTCAGG
	GSNOR-N-R	CCGCTCGAGTCAATTTGACCCTGGTTCTAC
	GSNOR-C-F	CGGGATCCGTTGCCATTTTCGGTCTTGG
	GSNOR-C-R	CGGAATTCTCATTTGCTGGTATCGAGGAC
	GSNOR-GST-F	CGGAATTCATGGCGACTCAAGGTCAGG
	GSNOR-GST-R	CGGAATTCTCATTTGCTGGTATCGAGGAC
	CaM4-BamH1-F	CGGGATCCATGGCGGATCAGCTAACTGA
	CaM4-EcoR1-R	CGGAATTCCTTAGCCATCATAATCTTGACA
RT-qPCR	CaM1-qRT-F	GATGATGATGACCTTTTTATGC

	CaM1-qRT-R	TTACACACAAAAGTCACAAACC
	CaM2-qRT-F	GATGGCAAAGTGAGGAAAC
	CaM2-qRT-R	ATGAAATTTGGGACGAATG
	CaM3-qRT-F	GGACTCGAGGTATGTTTTCTGCTT
	CaM3-qRT-R	TGTTCAGACGCAAAATAGAGCATAA
	CaM4-qRT-F	TCTACAAAAGAATGATTCGACC
	CaM4-qRT-R	TTATGCCAACGAGAAAGAAAC
	CaM5-qRT-F	ACAACTTCTTCGGCTTTCTC
	CaM5-qRT-R	TAACCAGCAAAAACCAGCC
	CaM6-qRT-F	AGCCCACAAAAAGAAACAAG
	CaM6-qRT-R	ATGTCCTAAAGAACGCAACC
	CaM7-qRT-F	TTTGTTGGTCGTGATTTTTTGG
	CaM7-qRT-R	CGTCACGGACAATAACAAAACC
	COR15A-qRT-F	CAACATCCTCGATGACCTCAA
	COR15A-qRT-R	GCTTCTTTACCCAATGTATCTGC
	GSNOR-qRT-F	ATCCTCGCACTCTCACTATCT
	GSNOR-qRT-R	GTGTGACAAAGAGCAGTGT
	KIN2-qRT-F	GCAATGTTCTGCTGGACAAG
	KIN2-qRT-R	GTTGACTCGGATCGCTACTT
	RD22-qRT-F	ACGTCAGGGCTGTTTCCAC
	RD22-qRT-R	TACTTCTGTTTGTGACACACC
	RD29A-qRT-F	TTCCGTTGAAGAGTCTCCAC
	RD29A-qRT-R	AACAAAACACACATAAACATCC
	RD29B-qRT-F	CCACGGTCCGTTGAAGAGTC
	RD29B-qRT-R	CAAAAACACAAACATTCAAAAGC
	18S-qRT-F	CGGCTACCACATCCAAGGAA
	18S-qRT-R	TGTCACTACCTCCCCGTGTCA
RT-PCR	CaM1RT-F	AAGGAAGAGAAGAAAGACGAT
	CaM1RT-R	GGTTAAACTCCTAGAAGCAT
	CaM4RT-F	AACCAAAGAAACGAGAAGAAG
	CaM4RT-R	AAACCCAAGACATTAAAGGTC
	ACTIN2RT-F	AGGCACCTCTTAACCCTAAAGC
	ACTIN2RT-R	GGACAACGGAATCTCTCAGC
RNAi construction	miCaM1-2-I	GATGGAAGTCGATAGTGCCGTTCTCTCTCTTTTGTATTCC
	miCaM1-2-II	GAGAACGGCACTATCGACTTCCATCAAAGAGAATCAATGA

	miCaM1-2-III	GAGACCGGCACTATCCACTTCCTTCACAGGTCGTGATATG
	miCaM1-2-IV	GAAGGAAGTGGATAGTGCCGGTCTCTACATATATATTCCT
	miCaM1/4-2-I	GATTAATCTTGACAAACTCCTAGTCTCTCTTTTGTATTCC
	miCaM1/4-2-II	GACTAGGAGTTTGTCAAGATTAATCAAAGAGAATCAATGA
	miCaM1/4-2-III	GACTCGGAGTTTGTCTAGATTATTCACAGGTCGTGATATG
	miCaM1/4-2-IV	GAATAATCTAGACAAACTCCGAGTCTACATATATATTCCT
